# The *Lancet* Commission on prostate cancer: planning for the surge in cases

**DOI:** 10.1016/S0140-6736(24)00651-2

**Published:** 2024-04-04

**Authors:** Nicholas D James, Ian Tannock, James N’Dow, Felix Feng, Silke Gillessen, Syed Adnan Ali, Blanca Trujillo, Bissan Al-Lazikani, Gerhardt Attard, Freddie Bray, Eva Compérat, Ros Eeles, Omolara Fatiregun, Emily Grist, Susan Halabi, Áine Haran, Daniel Herchenhorn, Michael S Hofman, Mohamed Jalloh, Stacy Loeb, Archie MacNair, Brandon Mahal, Larissa Mendes, Masood Moghul, Caroline Moore, Alicia Morgans, Michael Morris, Declan Murphy, Vedang Murthy, Paul L Nguyen, Anwar Padhani, Charles Parker, Hannah Rush, Mark Sculpher, Howard Soule, Matthew R Sydes, Derya Tilki, Nina Tunariu, Paul Villanti, Li-Ping Xie

**Affiliations:** Institute of Cancer Research, London, UK; The Royal Marsden NHS Foundation Trust, London, UK; Princess Margaret Cancer Centre, University Health Network, Toronto, ON, Canada; University of Aberdeen, Aberdeen, UK; University of California, San Francisco, USA; Oncology Institute of Southern Switzerland, Bellinzona, Switzerland; University of Manchester, Manchester, UK; The Christie Hospital, Manchester, UK; University College London, London, UK; MD Anderson Cancer Centre, Houston, TX, USA; University College London, London, UK; International Agency for Research on Cancer, Lyon, France; Tenon Hospital, Sorbonne University, Paris; AKH Medical University, Vienna, Austria; Institute of Cancer Research, London, UK; The Royal Marsden NHS Foundation Trust, London, UK; Lagos State University Teaching Hospital, Lagos, Nigeria; University College London, London, UK; Duke Cancer Institute, Durham, NC, USA; The Royal United Hospitals Bath NHS Foundation Trust, Bath, UK; Latin America Cooperative Group, Rio de Janeiro, Brazil; Peter MacCallum Cancer Centre, Melbourne, VIC, Australia; Université Cheikh Anta Diop de Dakar, Dakar, Senegal; New York University, New York, NY, USA; Manhattan Veterans Affairs, New York, NY, USA; University College London, London, UK; University of Miami, Miami, FL, USA; University College London, London, UK; Institute of Cancer Research, London, UK; The Royal Marsden NHS Foundation Trust, London, UK; University College London, London, UK; Dana Farber Cancer Institute, Boston, MA, USA; Memorial Sloan Kettering Cancer Center, New York, NY, USA; Peter MacCallum Cancer Centre, Melbourne, VIC, Australia; Tata Memorial Centre, Mumbai, India; Harvard University, Boston, MA, USA; Mount Vernon Cancer Centre, London, UK; University College London, London, UK; University College London, London, UK; University of York, York, UK; Prostate Cancer Foundation, Santa Monica, CA, USA; University College London, London, UK; Martini-Klinik Prostate Cancer Center and Department of Urology, University Hospital Hamburg-Eppendorf, Hamburg, Germany; Department of Urology, Koc University Hospital, Istanbul, Türkiye; Institute of Cancer Research, London, UK; The Royal Marsden NHS Foundation Trust, London, UK; Movember, Melbourne, VIC, Australia; First Affiliated Hospital, Zhejiang University School of Medicine, Hangzhou, China

## Abstract

Prostate cancer is the most common cancer in men in 112 countries, and accounts for 15% of cancers. In this Commission, we report projections of prostate cancer cases in 2040 on the basis of data for demographic changes worldwide and rising life expectancy. Our findings suggest that the number of new cases annually will rise from 1·4 million in 2020 to 2·9 million by 2040. This surge in cases cannot be prevented by lifestyle changes or public health interventions alone, and governments need to prepare strategies to deal with it. We have projected trends in the incidence of prostate cancer and related mortality (assuming no changes in treatment) in the next 10–15 years, and make recommendations on how to deal with these issues.

For the Commission, we established four working groups, each of which examined a different aspect of prostate cancer: epidemiology and future projected trends in cases, the diagnostic pathway, treatment, and management of advanced disease, the main problem for most men diagnosed with prostate cancer worldwide. Throughout we have separated problems in high-income countries (HICs) from those in low-income and middle-income countries (LMICs), although we acknowledge that this distinction can be an oversimplification (some rich patients in LMICs can access high-quality care, whereas many patients in HICs, especially the USA, cannot because of inadequate insurance coverage). The burden of disease globally is already substantial, but options to improve care are already available at moderate cost. We found that late diagnosis is widespread worldwide, but especially in LMICs, where it is the norm. Early diagnosis improves prognosis and outcomes, and reduces societal and individual costs, and we recommend changes to the diagnostic pathway that can be immediately implemented. For men diagnosed with advanced disease, optimal use of available technologies, adjusted to the resource levels available, could produce improved outcomes. We also found that demographic changes (ie, changing age structures and increasing life expectancy) in LMICs will drive big increases in prostate cancer, and cases are also projected to rise in high-income countries. This projected rise in cases has driven the main thrust of our recommendations throughout. Dealing with this rise in cases will require urgent and radical interventions, particularly in LMICs, including an emphasis on education (both of health professionals and the general population) linked to outreach programmes to increase awareness. If implemented, these interventions would shift the case mix from advanced to earlier-stage disease, which in turn would necessitate different treatment approaches: earlier diagnosis would prompt a shift from palliative to curative therapies based around surgery and radiotherapy. Although age-adjusted mortality from prostate cancer is falling in HICs, it is rising in LMICs. And, despite large, well known differences in disease incidence and mortality by ethnicity (eg, incidence in men of African heritage is roughly double that in men of European heritage), most prostate cancer research has disproportionally focused on men of European heritage. Without urgent action, these trends will cause global deaths from prostate cancer to rise rapidly.

On the basis of our assessments of the evidence base, we have identified and prioritised several recommendations for both immediate and long-term interventions to mitigate the current and projected future global impact of prostate cancer. We make detailed practical recommendations for the four highest-priority areas identified. First, diagnostic pathways should be modified to facilitate early detection of prostate cancer while avoiding overdiagnosis and overtreatment of trivial disease. These pathways should be linked to broader men’s health checks in LMICs in view of expected rises in diseases such as diabetes driven by the same demographic trends. The case for prostate cancer screening for all men aged 50–70 years (and all men of African origin aged 45–70 years) in HICs is strengthening with improved use of technologies such as MRI and growing evidence for the safety of active surveillance.

Second, novel methods of empowering patients, such as cloud-based medical record systems, should be exploited to enable doctors and patients to make informed, personalised case-management plans. Artificial intelligence systems could supplement deficits in health profession numbers and skills, especially—but not only—in LMICs. Such systems could not only already accurately diagnose cancers but also subdivide disease into potentially valuable additional subgroups to help with treatment selection. In environments with few or no pathologists, these changes could be transformational. In HICs, the additional information provided by artificial intelligence-supported diagnosis (eg, from rapid processing of large numbers of tissue sections) compared with conventional pathology alone also has huge potential to rapidly drive change. Giving control of records to patients can be an effective way to empower people. Linking cloud-based records to artificial intelligence systems could allow access to context-sensitive, up-to-date advice for both patients and health professionals, and could be used to drive evidence-based change in all settings. Clearly there are concerns about the potential risks of such systems, such as misinterpretation of data. However, in low-resource settings, the emerging evidence is that accuracy—for pathology, for example—is already high and improving. With careful implementation, artificial intelligence could contribute to improvements in quality of care, particularly in LMICs, in particular in the near future.

Third, resource-sensitive guidelines should be implemented to maximise the effect of available therapies, especially surgery and radiotherapy, use of which is often limited in LMICs. There is a linked urgent need for expansion of radiotherapy and surgery services (mainly in LMICs but also in some HICs with uneven provision), and, in view of the timelines for investment in equipment and training of staff, these changes need to be set in motion now to deliver projected future care needs. Similar considerations with regards to access and distribution apply to drug therapies: optimal use of available therapies in all settings could improve outcomes. Even where resources are adequate, consistent evidence suggests that application of best practice is variable.

In addition to these recommendations, research and the development of risk-stratified regulatory models need to be facilitated. Drug repurposing and dose de-escalation should be supported and studied. Novel clinical trial designs, such as multi-arm platforms, should be supported and expanded. Lessons should be learned from how low-cost HIV drugs were made available and distributed globally to better meet the needs of men with prostate cancer in LMICs. Additionally, the rapid roll out of studies of COVID-19 vaccines and therapies shows that effective, large-scale trial programmes are feasible and can lead to improvements in care. More research is needed into how disease prognosis, outcomes, and treatment effects (and side-effects) differ in different ethnic groups and socioeconomic settings.

The *Lancet* Commission on prostate cancer provides an agenda for a realistic programme of changes, which, if implemented, will improve the health of men globally both now and in future. The coming increases in prostate cancer are brought about by rising life expectancy and changes in population age structures. Unlike other large-scale problems, such as lung cancer or cardiovascular diseases, this rise in cases is not preventable by public health strategies. Nonetheless, the effects of the global rise in prostate cancer can be mitigated. The findings in this Commission provide a pathway forwards for healthcare providers and funders, public health bodies, research funders, governments, and the broader patient and clinical community.

## Introduction

Prostate cancer is the most common cancer by incidence in men in 112 countries (as of 2020), and accounts for one in every 14 cancers diagnosed globally, and 15% of all male cancers.^1–3^ Among men, the disease ranks second only to lung cancer in terms of cancer mortality.^3^

**Figure 1:**
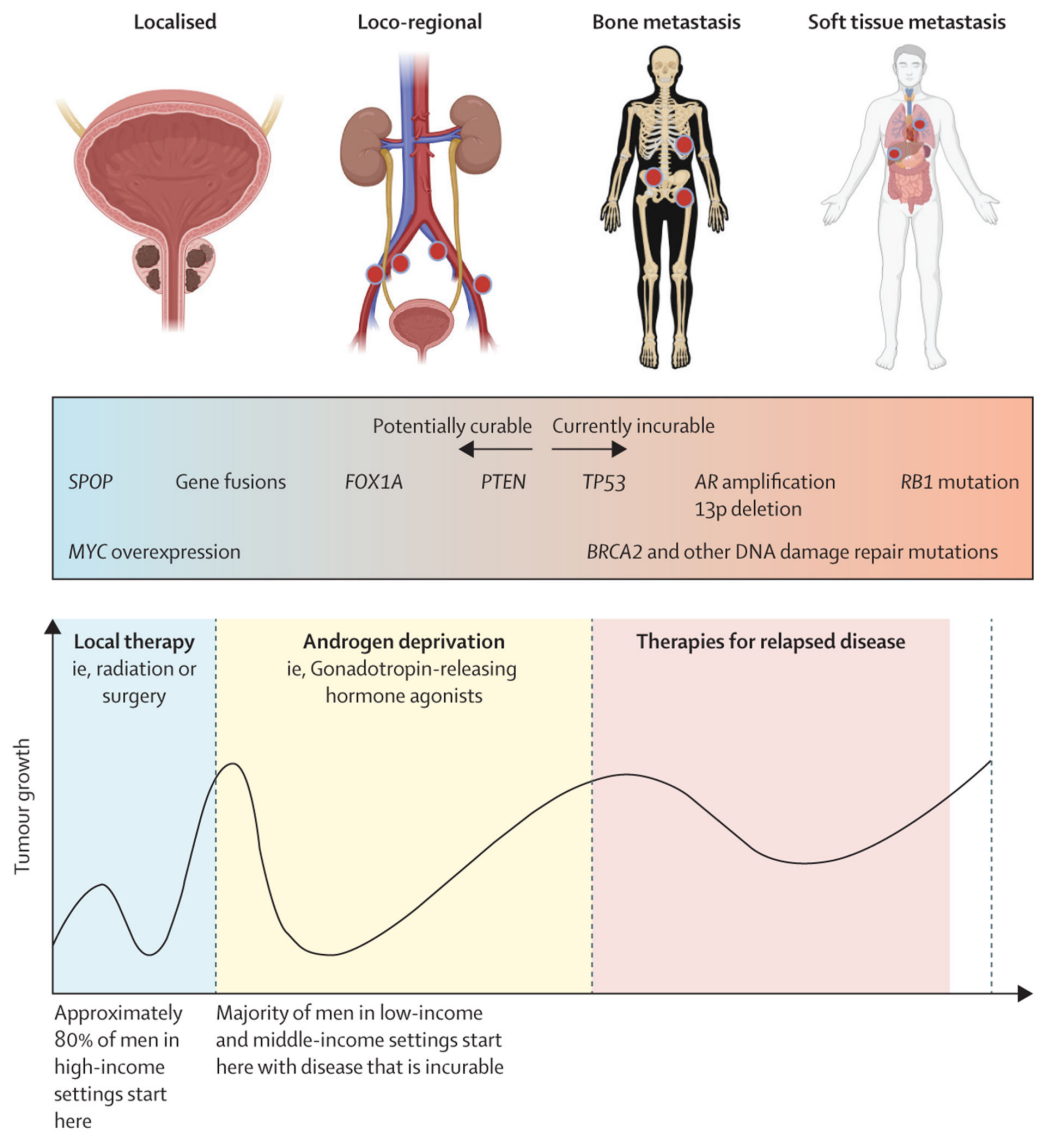
Overview of prostate cancer staging and biology

The prostate gland is situated at the base of the bladder, and its basic function is to provide the seminal fluid that nourishes and supports spermatozoa in transit from the testicles during ejaculation. Cancers arise in the lining epithelium of the prostate gland: they range from low-grade tumours that require no treatment to rapidly growing, highly lethal cancers. The disease pathways and some of the underlying biology and genetics are summarised in figure 1.^4^ The biology underlying this broad range of cancer behaviour, from indolent to highly lethal, is only partly understood. Overdiagnosis of clinically trivial disease potentially leads to overtreatment and harm, whereas late diagnosis of potentially lethal disease is a major, and growing, cause of morbidity and premature death, particularly in low-income and middle-income countries (LMICs).^1,5^ Treatment of prostate cancer is determined by stage (ie, TNM tumour classification) and tumour grade (ie, Gleason grading), and also depends on PSA concentrations and the age and broader health of the patient. Gleason grading goes from 1 (low) to 5 (high), although scores of 1 and 2 are now rarely used, and 3 is the lowest grade in clinical use (the system has purposefully not been updated to enable back comparison to historical series). Cancers are assigned two Gleason scores: the majority score and the score for the main minor grade. Cancers can be considered clinically insignificant if at least two of these three criteria are met: the Gleason scores are 3 and 3 (or 3 and 4 if the majority grade is 3); PSA concentrations are less than 10 ng/mL; and the cancer is classed as T1 or T2N0M0 per TNM. All other tumours should be considered clinically significant and therefore to require treatment (dependent on age, fitness, and comorbidities). If a man has comorbidities and a short life expectancy, even rapidly growing tumours can be monitored or offered minimal treatment.^6^ Conversely, relatively slow growing tumours in fit, young patients might warrant treatment to prevent harm later.

In this Commission, we make projections of future trends in cases of prostate cancer, identify the best approaches to diagnosis and management in different health-care settings, and make recommendations for evidence-based policy and clinical practice, research, and investment. We seek to empower not only health-care professionals and providers but also patients and their families to act as agents for change. Advances in imaging (particularly prostate-specific membrane antigen [PSMA] PET and CT), molecular and genetic technologies, high-precision radiotherapy for curative treatment, and new therapies for advanced disease, along with the use of artificial intelligence (AI) tools, are likely to have an increasing role in detection and management of prostate cancer. Many of these technologies are potentially scalable, affordable, and available in LMICs, but a major challenge is identification of optimal strategies for deployment.

Our work builds on key findings from previous *Lancet* Commissions, in particular those on access to radiotherapy^7,8^ and surgery.^9^ To research and prepare this Commission, we formed four working groups focusing on different areas across the disease spectrum: epidemiology and future projected trends in prostate cancer cases, the diagnostic pathway, treatment, and management of advanced disease. The resulting Commission report, which is split into seven parts, gives an overview of current and future problems, and provides recommendations for change in both policy and practice based on our projections of a rise in cases of prostate cancer (mainly in LMICs). The Commissioners include ethnically and geographically diverse global experts in medical and radiation oncology, urological surgery, epidemiology, health economics, statistics, and genetics, whose work was supported by UK-based early career researchers in prostate cancer. We sought input from a broad range of global sources, including regulators such as the US Food and Drug Administration and patient advocate organisations, such as Movember.

## Part 1: Contemporary and future scale and distribution of the prostate cancer burden

We assessed contemporary global variations in the incidence and mortality of prostate cancer in 185 countries or territories by using the International Agency for Research on Cancer’s (IARC) Global Cancer Observatory estimates of the burden in 2020 (hosted on their the Global Cancer Observatory).^1,2^ The data sources and methods used in compiling national estimates have been described previously.^10^ Briefly, the Global Cancer Observatory estimates are assembled at the national level on the basis of the best available sources of cancer incidence (eg, national or subnational cancer registries) and mortality data (eg, national vital statistics). High-quality cancer registry data are available in only around a third of countries, and high-quality mortality data are available in only a quarter of countries,^11^ which means that global prostate cancer estimates—and particularly those for countries without high-quality data—should be interpreted with caution. To examine temporal trends in incidence, we used data spanning at least 10 years of data from 1975 onwards for 31 countries (based on the data compiled in the IARC’s Cancer Incidence in Five Continents series),^3^ and assessed trends in prostate cancer mortality for the same 31 countries by using population-based data from the WHO mortality database.^12^ Cases of prostate cancer data were identified based by code C61 from the tenth revision of the International Classification of Disease.

We also predict the number of prostate cancer cases and deaths in the year 2040 by incorporating trends-based scenarios for 21 standardised UN regions used by IARC for epidemiological modelling (details of methods in Ferlay and colleagues).^13^ The predictions, which were developed using IARC’s Global Cancer Observatory,^13^ take into account both projected demographic changes (population ageing and population growth) and temporal trends in incidence and mortality.^13–15^ These trends-based burden predictions were derived at the regional level on the basis of recent temporal developments within constituent countries, and we assume constant trends from 2020 to 2040. Our final estimates are thus based on likely percentage changes in incidence and mortality rates per annum at the world regional level. However, as with all estimates, these should thus be interpreted with caution. However, it should also be noted that in poorer countries, many people will die without any diagnosis being made or, if made, recorded. Hence, all estimates will be likely less than the true incidence with perfect data.

**Figure 2:**
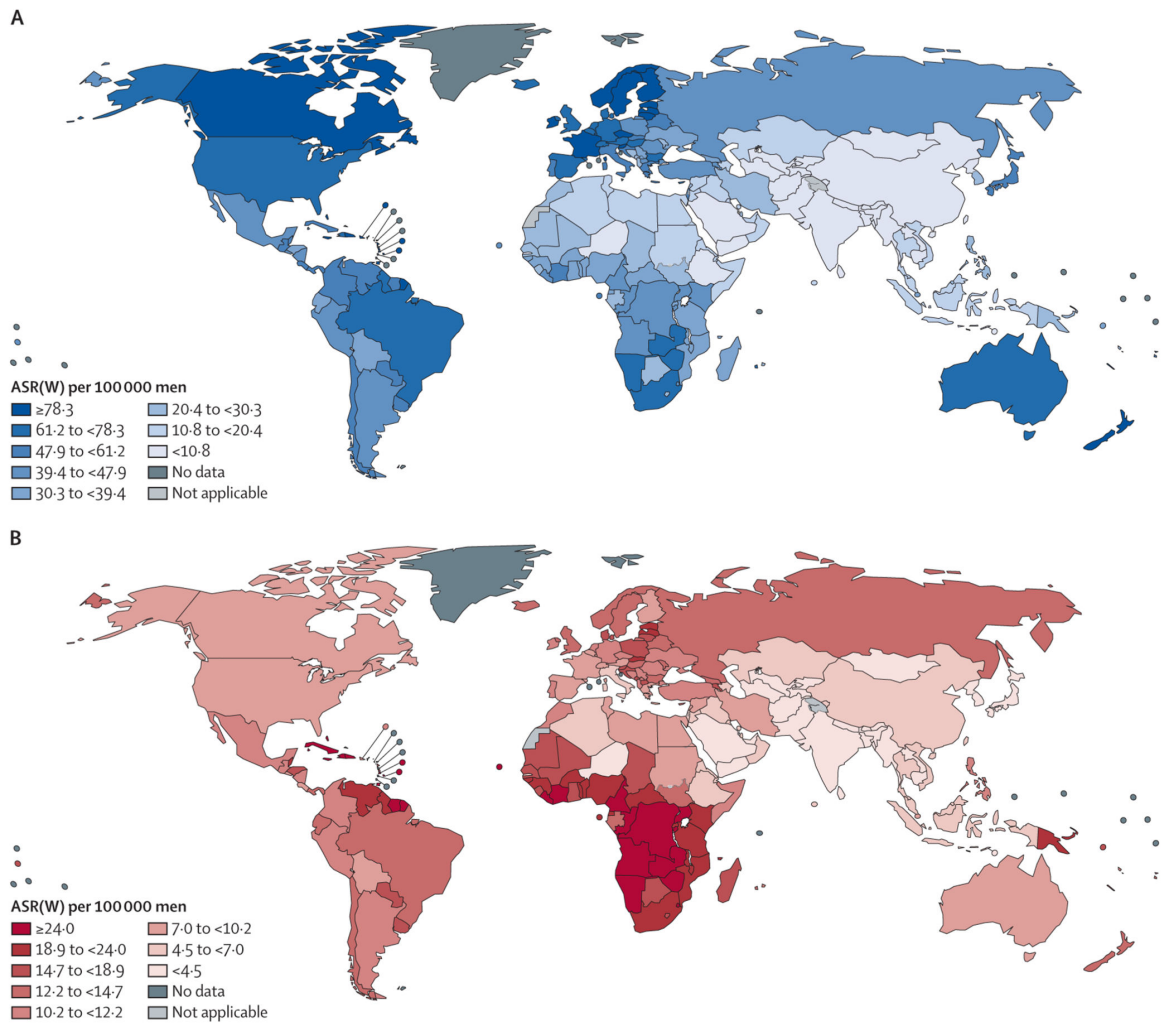
Global variations in prostate cancer incidence (A) and mortality (B), 2020 ASR(W)=age-standardised incidence rates.

**Figure 3:**
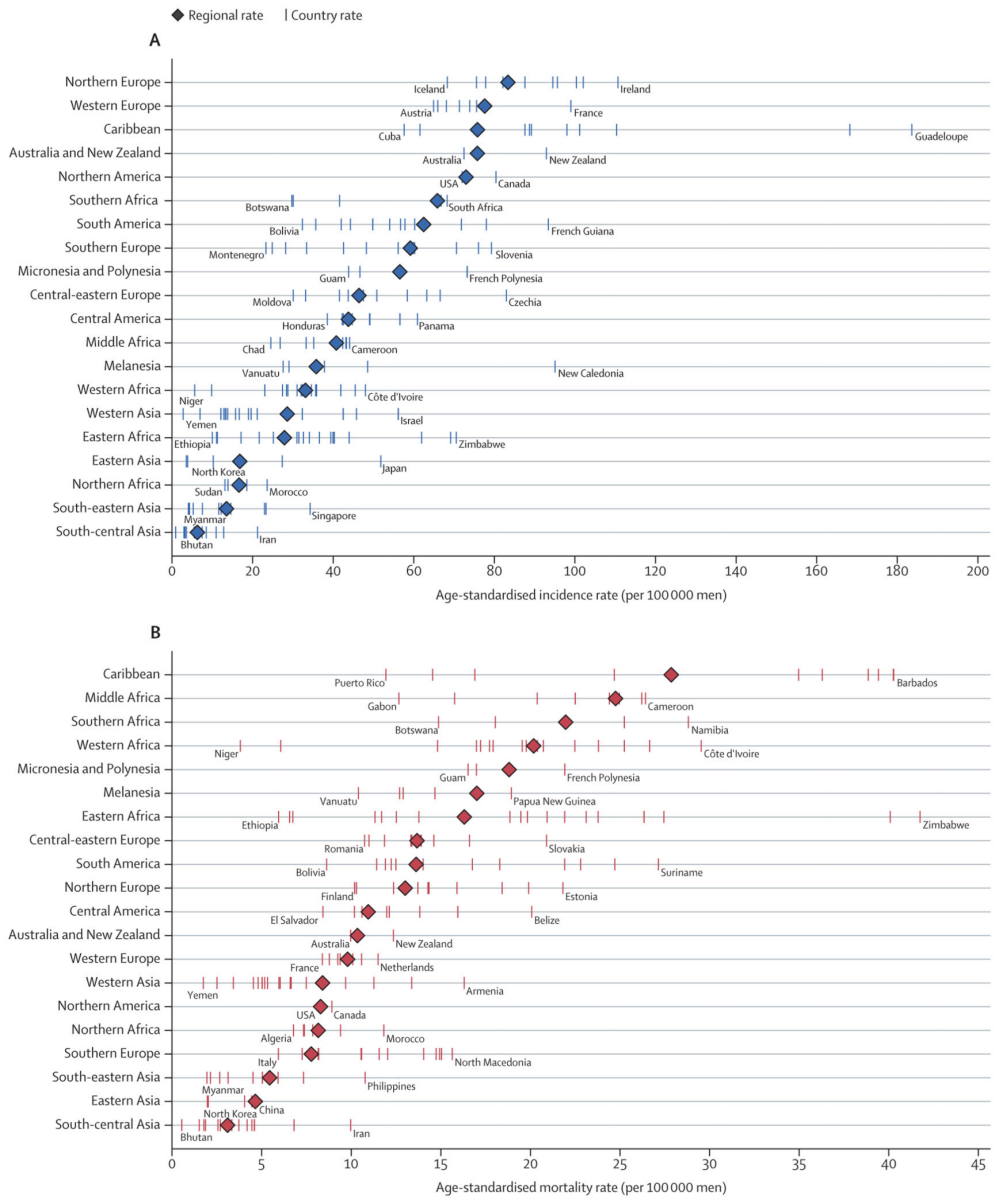
Regional variations in prostate cancer incidence (A) and mortality (B), 2020 Minimum and maximum national rates in each region are shown.

There were an estimated 1·4 million new cases of prostate cancer in 2020. Incidence varies substantially globally, with the highest incidence in northern and western Europe, the Caribbean, Australia, New Zealand, North America, and southern Africa (figure 2).^5,16^ National incidence can differ by a factor of four even within these regions (figure 3). Incidence is lower in most parts of Africa and across Asia, although there are large inter-country variations. The large variations in the recorded incidence of prostate cancer between countries are partly related to health-system factors linked to social and economic development and diagnostic practices such as the extent to which prostate-specific antigen (PSA) is used.^5^ The underlying variations in incidence are poorly understood because most research into the genetics and epidemiology of prostate cancer has been done in White populations. However, some underlying risk factors have been identified. Incidence is particularly high among Black men in the USA and the Caribbean, for example, which suggests a potential link between west African ancestry and increased risk of prostate cancer.^17,18^ Established risk factors include advancing age, family history, some genetic mutations (eg, in *BRCA1* and *BRCA2*), and inherited disorders such as Lynch syndrome. Few lifestyle and environmental factors have been identified, although smoking, excess bodyweight, and nutritional factors could potentially be linked with increased risk of prostate cancer.^19^

Prostate cancer accounted for around 375 000 deaths worldwide in 2020,^1,2,10^ making it the fifth leading cause of cancer death among men. National patterns in mortality bear little relation to those for incidence (figures 2, 3),^2,5,10^ other than in the Caribbean, which has the highest mortality in the world. Mortality is particularly high in sub-Saharan Africa, Micronesia, and Polynesia. Discrepancies between the recorded incidence and mortality are probably driven by difference in coverage of PSA testing (which substantially affects incidence—lower rates of asymptomatic testing of PSA will generally lead to increased rates of late diagnosis, as occurred in the USA when directives were issued restricting access to PSA testing^20^), the availability of effective therapy, social and environmental stressors (such as the need to travel long distances to access services), and the proportion of the population that is of west African ancestry.^18,21,22^

**Figure 4:**
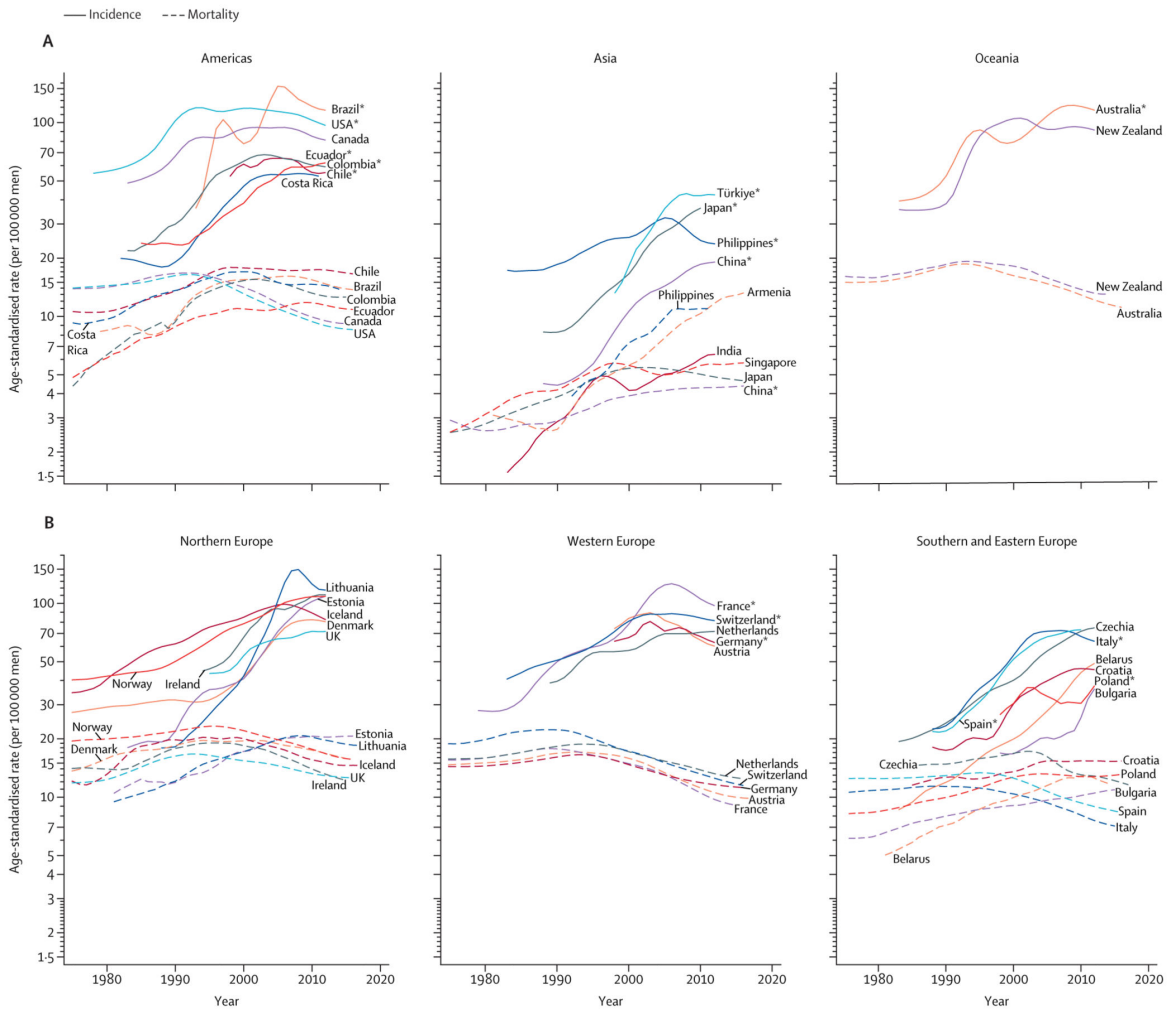
Temporal trends in prostate cancer incidence and mortality in countries in the Americas, Asia, and Oceania (A) and Europe (B) Solid lines represent incidence, whereas dashed lines represent mortality. Note the use of semi-logarithmic scale. The trend lines have been smoothed by using Loess regression. *Data for these countries come from regional registries.

Temporal trends in incidence and mortality by region are presented in figure 4. In the USA, Canada, and Australia, the recorded incidence increased rapidly in the late 1980s and early 1990s because of the widespread introduction of PSA testing, which allowed for early detection of prostate cancers, including clinically insignificant cases that would otherwise not have been diagnosed. These rapid increases in incidence were followed by sharp reductions, probably reflecting depletion of the number of prevalent latent cancers in the general population and subsequent reductions in PSA testing.^23,24^ In other countries, including several in Europe, less marked but similar patterns were noted, suggesting a later and more gradual adoption of PSA testing (figure 4). By contrast, the incidence of prostate cancer continues to increase in China and eastern Europe (figure 4). Rapid increases in incidence of 2–10% per year were noted in sub-Saharan Africa between 1995 and 2018, which could reflect increased awareness of prostate cancer and improvements in health-care systems (with broader use of PSA testing and possibly of transurethral resection).^14^ Many patients in sub-Saharan Africa are undertreated and their disease is insufficiently staged.^25^ As a result, survival is poor among these patients relative to the global average, which highlights the need for better diagnostic workups, earlier diagnosis, and increased access to care in sub-Saharan Africa.^25^

Mortality rates for prostate cancer have decreased since the mid-1990s in North America, Oceania, and northern and western Europe (figure 4), probably because of advances in treatment and early detection. During the same period, mortality rates increased in many countries in Asia, Africa, and central and eastern Europe, probably due to a combination of increased incidence and insufficient access to effective treatment.^5^ In the USA, the diagnosis of regional and advanced-stage prostate cancer has increased since 2010, with a concomitant increase in advanced-stage mortality from 2012.^20^ Although overall mortality from prostate cancer is lower in Asia than in other regions (figures 2, 3) for reasons that are poorly understood, the same rises in incidence and mortality over time are apparent, particularly in China.

Global differences in mortality are partly due to economic factors but are also influenced by ethnicity. Incidence of and mortality from prostate cancer differ between Black and White people grobally.^17^ In the UK, which has universal health coverage, whether the higher mortality among Black populations is driven by differences in incidence, differing disease biology, or external factors such as deprivation or racism is unclear.^26^ Data for how racial differences in disease biology affect treatment outcomes (as opposed to disease incidence) are scarce, but, when available, suggest that Black men might have better outcomes than White men in some treatment contexts—eg, post-relapse chemotherapy.^21^ However, any ethnic differences in biology are, at present, insufficient to drive differences in therapy. Although issues such as social deprivation drive differences in most health outcomes, specific data for these effects in the context of prostate cancer have not been reported.

**Figure 5:**
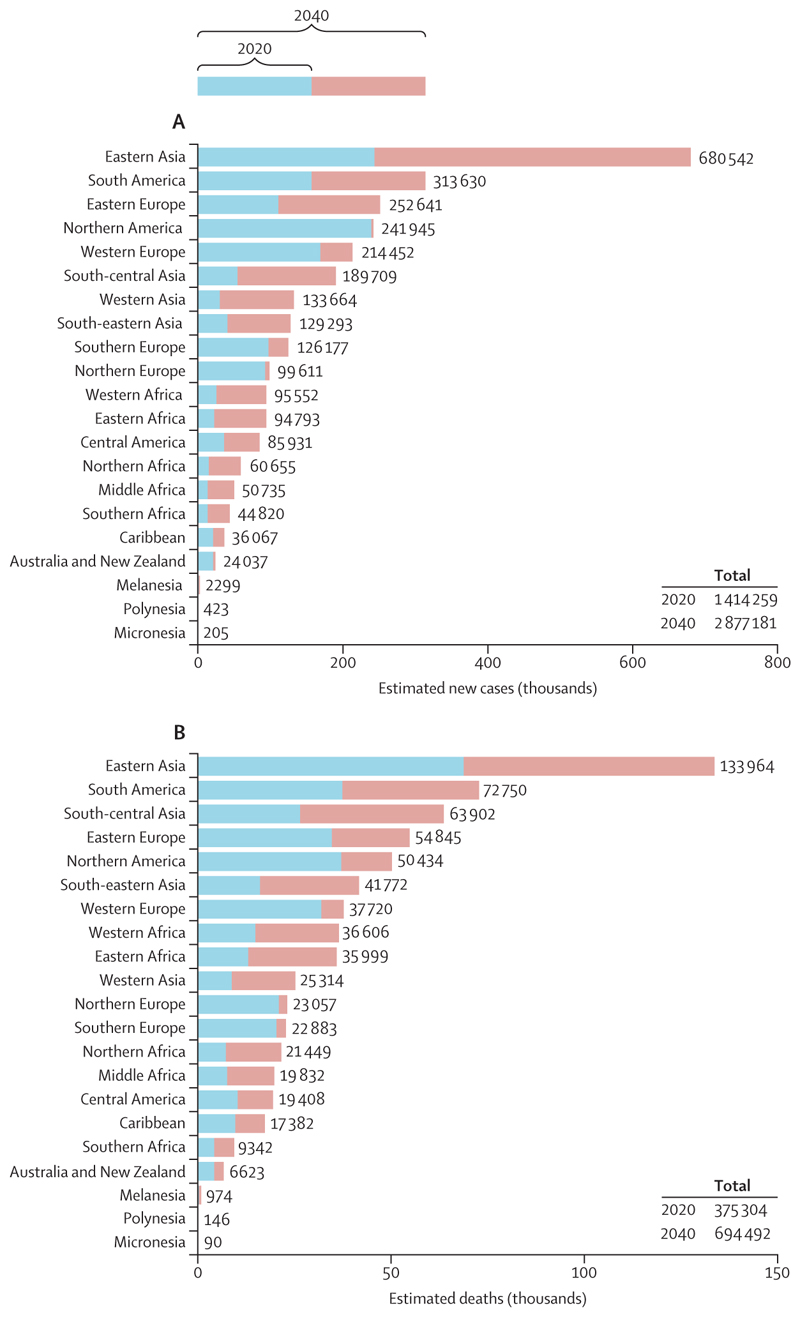
Estimated number of new cases of (A) and deaths from (B) prostate cancer among men and boys aged 0–85 years in 2020 and 2040, by UN world region Predictions to 2040 take into account national population projections and regional trends in prostate cancer incidence and mortality rates based on recent reports.^14,15,27,28^ Custom annual percent changes have been applied for both incidence (Northern America –1%, Eastern Asia 2%, Eastern Africa 3%, Middle Africa 3%, Northern Africa 3%, Southern Africa 3%, Western Africa 3%, Caribbean 0·5%, Central America 1%, South-eastern Asia 2%, South-central Asia 3%, Western Asia 3%, Eastern Europe 3%, Northern Europe –1%, Australia and New Zealand –1%, and South America: 0·5%) and mortality (Northern America –1·5%, Eastern Asia –0·5%, Eastern Africa 1%, Middle Africa 1%, Northern Africa 1%, Southern Africa 1%, Western Africa 1%, Central America –0·5%, South-eastern Asia 0·5%, South-central Asia 1%, Western Asia 0·5%, Eastern Europe 0·5%, Northern Europe –2%, Southern Europe –1·5%, Western Europe –1·5%, Australia and New Zealand –1%, and South America –0·5%).

After accounting for projected demographic changes (such as increasing numbers of older men because of improved life expectancy plus changing age structures meaning that older people account for an increased proportion of the population) and assuming that reported trends in population-based incidence^13–15,27^ will continue to 2040, we predict that prostate cancer incidence will double from 1·4 million new cases per year in 2020 to close to 2·9 million cases per year in 2040 (figure 5A). A close to 3% global decline in incidence rates from 2020 to 2040 would be required for case numbers in 2040 to remain the same as those in 2020. Correspondingly, we estimated that prostate cancer deaths will rise by 85%, from 375 000 in 2020 to close to 700 000 by 2040 (figure 5B).

### Towards better prostate cancer surveillance and research

Global assessment of the patterns and trends of cancer worldwide is hampered by poor availability of high-quality data, especially in LMICs. Led by IARC, the Global Initiative for Cancer Registry Development is a partnership of leading cancer organisations that is seeking to improve the availability of robust cancer incidence data. Since 2012, six IARC regional hubs for cancer registration have been established covering Africa, Asia, Latin America, the Caribbean, and the Pacific Islands. These hubs provide technical assistance to registries through a so-called train-the-trainer approach, with site visits to countries to support local surveillance plans. This IARC initiative has the potential to improve the accuracy of global cancer estimates, and to provide governments with the local data needed to prioritise and assess cancer control activities to reduce cancer morbidity and mortality.^29^

Nonetheless, despite the limitations of the available data, some clear differences in incidence and mortality can be observed. To highlight some of the drivers of these differences, we will focus on two examples: men of west African descent (panel 1) and men in India (panel 2). The former is important given the very high incidence of prostate cancer in men of west African heritage. Experience in India is important because, although incidence is proportionally lower there than in west Africa, the population is large and life expectancy is improving, which mean that absolute numbers of older men are rising. The Indian health-care system is also a microcosm of the wider world, with a relatively wealthy middle class getting high-quality care and a large, poorer population experiencing problems in access to care typical of LMICs.

### Conclusions

Prostate cancer is already a major cause of morbidity and mortality worldwide, and the numbers are predicted to double by 2040. The true number of cases are likely to be higher than the recorded figures because of under-diagnosis and poor reporting, particularly in LMICs. The relatively greater population ageing and growth in LMICs (compared with HICs) will lead to even larger increases in prostate cancer in the coming years, causing suffering and economic hardship. The same demographic factors that are driving increases in the incidence of prostate cancer will also lead to parallel increases in other diseases of older populations, which will all need to be dealt with by health-care systems. With better planning, many of these harms can be substantially mitigated. Solutions with broad applicability are urgently needed. Some potential solutions, which could include changes to diagnostic and treatment pathways, are discussed later in this Commission.

## Part 2: The diagnostic pathway

The landscape of prostate cancer differs substantially around the world, and therefore so too do the priorities for prostate cancer detection. In LMICs, prostate cancer is mostly diagnosed at an advanced age in people with high total serum PSA concentrations, locally advanced disease, or metastasis (or all three).^34^ Early detection is more common in HICs, driven by higher rates of PSA testing and better access to treatment. This high frequency of early detection translates into lower mortality rates per incident case than is observed in LMICs.^15^ There is a strong correlation between the Human Development Index of a country and risk of death from prostate cancer.^15^ Underdiagnosis and undertreatment cause harm in LMICs; overdiagnosis and overtreatment lead to different harms in HICs. These opposing issues both need to be addressed.

Global variations in the incidence of prostate cancer are probably partly associated with differential risk profiles, but differences in use of PSA testing and levels of awareness of potential signs and symptoms of prostate cancer are also important contributory factors. An improved understanding of individual risk would account for ethnicity, advancing age, family history of prostate cancer, and genetics (eg, mutations in *BRCA1, BRCA2, ATM*, and the mismatch repair genes). The most frequent inherited abnormality in prostate cancer is *BRCA2*, which seems to be particularly key in driving poor prognosis in monogenic risk-linked cancers.^35–37^ Polygenic risk scores will probably also be of value in this context: the contribution of monogenic disorders, although important, accounts for only a proportion of the excess risk in people with strong family histories of prostate cancer.^38^ Emerging data suggests that the highest risk quintile of polygenic risk scores accounts for almost 50% of incident cases of prostate cancer.^39^ Such scores could be used to target screening and prevention services to men at high risk of prostate cancer. However, polygenic risk scores are only one aspect of screening and prevention programme. There are increasing opportunities to refine a risk-adapted diagnostic pathway to ensure early detection of clinically significant prostate cancer (before progression, metastasis, and negative effects on quality of life and life expectancy) while avoiding overdiagnosis of low-grade disease (thereby preventing iatrogenic harm and unnecessary consumption of resources). The need for global consensus on a risk-adapted early detection pathway is further supported by the disconnect between incidence and mortality reported worldwide discussed in the previous section.

### Population-based screening for early detection

Screening for prostate cancer is a divisive topic. In the past, the term screening has been used to describe population-based screening of asymptomatic men. Publication of the initial results of a large European trial^40^ of prostate cancer screening and a more general cancer screening trial^41^ in 2009 led some expert groups (eg, the UK National Screening Committee, as recently as 2020)^42^ to conclude that population-based screening of all men by measuring PSA concentrations and performing digital rectal examinations was not justified and led to overdiagnosis and overtreatment. However, another European trial^43^ suggested a 21% improvement in prostate cancer-specific mortality with general population-based screening of all men versus no such screening, and as trial follow-up has increased, the rate of overdiagnosis has fallen. The number of people you need to screen to diagnose one case of prostate has decreased from 48 at 9 years’ follow-up to 18 at 16 years’ follow-up, and the number needed to screen to prevent a prostate cancer death has decreased to 570.^43^ Nonetheless, PSA-based screening of the general population is still viewed negatively, typified by the recommendation against PSA testing issued by the US Preventive Services Task Force in 2012, which was associated with a decline in prostate cancer diagnoses in North America^44,45^ and elsewhere.^46^ Public attitudes to PSA testing vary, and in HICs the highest rates of testing are in older, more affluent men.^47^ Attitudes among doctors vary hugely, especially between urologists, as evidence by professional bodies like the European Urology Association being in favour^48^ of testing while public health bodies like the US Preventive Services Task Force are opposed. This decrease in diagnoses was followed by a 6% increase in the number of patients with metastatic prostate cancer,^20^ suggesting that PSA-based screening might have been reducing the incidence of metastatic disease.

Concerns about overdiagnosis and overtreatment continue to dominate the debate surrounding PSA-based screening in HICs. In 2017, the US Preventive Services Task Force issued an updated recommendation that men aged 55–69 years should be counselled about the benefits and harms of PSA screening because testing might be associated with a small survival benefit (a grade C recommendation).^49^ However, because advanced age is such a strong risk factor, voluntary screening programmes, which are common in HICs, can result in over-testing in men aged 70 years or older and under-testing in men aged 50–70 years.^47^ Such under-testing is especially likely in younger men from ethnic minorities or deprived backgrounds—precisely the groups at highest risk of presentation with advanced disease and with the most life years to lose.^47^ Substantial regional variations linked with patient-requested screening have also been noted—eg, the proportion of patients with advanced disease at presentation in the UK varies from 12·5% in London to 30% in Scotland.^50^ An individualised risk-adapted early-detection strategy is now recommended in international prostate cancer guidelines.^51^ In the European Association of Urology’s PRAISE-U project, EU member states are comparing their different approaches to prostate cancer screening.^52^ All guidelines recognise the risk of overdiagnosis and the need to break the link between diagnosis and overtreatment without compromising the benefits that early detection could have for men with clinically significant prostate cancer.^43,51^

A key limitation of most trials of prostate cancer screening is that they have been done in HICs, where advanced disease at diagnosis is uncommon and efficient diagnostic tools and effective treatment are available (although, as noted, advanced disease rates vary hugely both within countries and by ethnicity and deprivation level^50^). Most screening trials have also largely been done in majority White populations in European and North American regions, and White people are often heavily over-represented in such studies^6,53^—a further major limitation. The efficacy of population-based PSA testing in LMICs, where most men present with late-stage disease, has never been assessed, and there is an urgent need for trials in these countries.

As discussed in detail later in the Commission, increased use of MRI in the diagnostic pathway reduces the risk of overdiagnosis of indolent disease.^32,54,55^ Nonetheless, some men will still be diagnosed with clinically insignificant disease. A key aspect of expanding the diagnostic pathway is that it must be linked to increased uptake of active surveillance in those diagnosed with indolent disease (see Part 3). Doing so will help to reduce over-treatment.

### Non-PSA biomarkers and genetic testing

Moderately raised PSA concentrations have low specificity for underlying prostate cancer, with a positive predictive value of 25–40% for PSA concentrations of 4–10 ng/mL; hence, more accurate biomarkers are needed.^56^ The discovery of novel biomarkers has enhanced diagnostic capabilities, leading to improved risk stratification with a reduction in unnecessary biopsies and improved detection of clinically significant prostate cancer.^57^ Various tests and measures have been developed, including the Prostate Heath Index, measurement of PCA3 concentrations, the 4Kscore test, MyProstateScore, Select mdxx, and ExoDX, but none of them has attained widespread acceptance for clinical utility.^51^ These tests need to be incorporated into multivariable clinical diagnostic models to better stratify patients’ risk, but predictive and comparative studies are needed that include men from a wider demographic spectrum (these tests have mostly been studied in White, high-income populations).^57^

As a result of the discovery that mutations in *BRCA1* and *BRCA2* predispose to development and aggressiveness of prostate cancer,^35^ germline testing for *BRCA1, BRCA2*, and other cancer predisposition syndromes such as Lynch Syndrome is likely to gain more prominence. Germline testing is not part of routine clinical practice anywhere globally, but could make a meaningful contribution to the diagnostic pathway.^51^ It could be appropriate in men with a family history of high-risk or metastatic prostate cancer, or a family history of cancer in general, and in men with multiple family members who were diagnosed with prostate cancer at a young age. However, monogenic risk profiling of the general population is likely to be of restricted value if done in isolation and profiling of multiple genetic alterations that alone confer small increases in risk (so-called polygenic risk scores) might be more effective. Linking polygenic risk assessment to targeted screening might improve detection of clinically significant disease while avoiding the costs and drawbacks of blanket screening. Trials of the incorporation of polygenic risk score as an additional indicator of risk are at the design stage. Research is aiming to establish whether cancers occurring in individuals at high genetic risk (eg, *BRCA2* carriers, those with high polygenic risk scores) are more aggressive than those in people with no detectable genetic predisposition. If this were shown to be true, it would reinforce the case for DNA profiling as part of a screening strategy, especially in HICs. On a population level, testing for *BRCA1, BRCA2*, and other cancer predisposition syndromes will also help to identify individuals at high risk of other cancers, such as breast and ovarian cancer.^58^

At present, there is little role for using circulating tumour DNA (ctDNA) in the diagnostic pathway, since only advanced cancers tend to produce concentrations that can be detected with present technologies.^59^

### MRI-based early detection

Use of multi-parametric MRI (mpMRI) in the triage pathway for men undergoing early detection, which is part of recommended practice in the UK and the EU, has been associated with substantial benefits. A 2019 Cochrane review^32^ suggested high mean sensitivity (0·91–0·95) for detection of clinically significant tumours, although mean specificity was substantially lower (0·35–0·37). MRI-based diagnosis has high negative predictive value and sensitivity for clinically significant cancers, which enable reliable ruling out of clinically insignificant cancers without the need for biopsies.^60^ Similar negative predictive values have been reported for MRI-based diagnosis outside formal trial settings, despite heterogeneities in disease prevalence, MRI scanners, and expertise of radiologists, urologists, and pathologists.^61–63^ The PROMIS^54^ and PRECISION^55^ studies have shown that systematically doing biopsies in all men in whom prostate cancer is suspected without use of pre-biopsy mpMRI leads to overdetection of clinically insignificant prostate cancer (ie, that are judged not to require treatment based on Gleason scoring, PSA concentrations, and tumour stage) and under-detection of clinically significant prostate cancer. These trials and others have led to guidelines (eg, from the European Association of Urology^51^), endorsing the use of mpMRI to guide the selection of patients to undergo prostate biopsy.

As a result, many patients in whom prostate cancer is suspected on the basis of raised PSA concentrations or the results of digital rectal examination could avoid undergoing biopsy (and a diagnosis of clinically insignificant prostate cancer) if their mpMRI results are normal. Use of mpMRI resulted in biopsy avoidance in 37% of men and reduced detection of clinically insignificant prostate cancers by 9% in an outpatient population in which roughly one in three cancers was judged clinically significant.^64^ However, when disease prevalence is higher and later diagnosis more common, the magnitude of benefit from use of mpMRI compared to transrectal ultrasonographically guided biopsy is smaller. In the PAIREDCAP study of MRI-targeted biopsy versus systematic biopsy,^65^ in which the prevalence of higher-grade cancers was substantially higher than the pooled prevalence in the Cochrane review^60^ of the same topic (61% *vs* 28%), the detection of higher-grade cancers by systematic biopsy was 15% compared with 8% in the Cochrane review. These findings suggest that, when there is an increased risk of clinically significant prostate cancer, a negative mpMRI result should not be used as evidence that a biopsy does not need to be taken.

The increasing use of AI could help to improve MRI by reducing scan times, minimising variance in interpretations between radiologists,^66^ automating the out-lining of the prostate gland, and identifying intraprostatic targets before biopsy and treatment. It could also enable diagnostic predictions of probable histology.^67^ In addition, research into simplification of the diagnostic sequences used in MRI, linked with initial AI-based interpretation, could facilitate increased throughput, reduce costs, and improve consistency.

In 2021, the European Association of Urology and the European Commission issued a new position paper on PSA screening and the role of MRI.^48^ Europe’s Beating Cancer Plan proposes that the EU Council changes its recommendation on cancer screening to include population-based screening for prostate cancer (both PSA testing and MRI). The European Urology Association’s 2022 guidelines^51^ on prostate cancer (produced in collaboration with a range of other professional bodies) and Europa UOMO (a European patient organisation) have endorsed this change of policy. The position on PSA screening in the USA remains unchanged since the US Preventive Services Task Force recommendations in 2012.

The Commissioners’ view is that unselected wide-scale population screening is not justified in HICs. However, targeted PSA testing, linked to MRI to reduce overdiagnosis, in men aged 50–69 years at high risk of disease is warranted in HICs. Asymptomatic PSA testing in older men is ineffective and leads to harm. PSA testing needs to be linked to MRI-based assessment and treatment only for high-grade tumours, which would mitigate overdiagnosis and overtreatment. Whether population-based prostate MRI screening, analogous to mammography for breast cancer, can be clinically effective and cost-effective is being assessed in studies such as Goteborg-2.^68^ This study is testing three different screening approaches: PSA-based only (concentrations ≥3 ng/mL prompt biopsy), PSA concentrations of at least 3·0 ng/mL and abnormal MRI results are necessary to do a biopsy, or PSA concentrations of at least 1·8 ng/mL and abnormal MRI results prompt biopsy. We eagerly await the results of this and similar studies. Programmes of early diagnosis are also urgently needed in LMICs.

### Addressing the gap in pathology provision

Pathology has a fundamental role in diagnosis, prognosis, and management decisions in prostate cancer. There is an urgent need for standardisation of pathology services, from sample processing to histopathology reporting, with clear demonstration of reproducibility and consistency. According to the experience of the Commissioners, poor management of samples (eg, inadequate identification or preservation with suitable fixatives), problems with collection and transportation, mislabelling, and lost samples are especially challenging in LMICs. Management of samples with automatic identification is needed to reduce errors and speed up reporting time. We suggest that radio frequency identification systems, commonly used in smart identification cards and for stock control in supermarkets could facilitate audits in the chain of processing of a given sample.^69^

There is a chronic worldwide shortage of pathologists in both HICs and LMICs. A 2019 report showed a 17·5% decrease in the number of pathologists in the USA between 2007 to 2017, with a corresponding 41·7% rise in diagnostic workload per pathologist.^70^ In the UK, only 3% of National Health Service (NHS) histopathology departments have enough staff to meet clinical demand.^71^ Although innovations like digital pathology, virtual microscopy, and AI promise improvement in pathology reporting (especially in LMICs and remote and rural settings), at present their use is largely restricted to research contexts. Potential benefits include online reporting (especially for patients in remote areas), and improved connection between centres for cases that need to be referred and reviewed in multidisciplinary team meetings. As for human pathologists, digital technologies and AI systems need to be benchmarked against accepted international standards such as those provided by the International Society for Urological Pathology.^72^

AI in particular has many potential benefits in prostate cancer diagnosis, but various issues need be overcome before these benefits can be reaped, including how best to enable high-throughput preparation of digital slides and handling of large datasets, technical problems related to tissue artifacts and complicated patterns of prostate cancer pathology, and the high upfront cost of slide scanners and software. Cost could limit the use of AI-based diagnosis in LMICs, unless the distributors of equipment are willing to adapt costs to the countries’ financial circumstances. There could also be legal considerations (eg, surrounding data protection) associated with the use of computer-aided diagnostics, and the effects of human biases on computer-aided workflows in prostate cancer have yet to be studied.

Despite these potential issues, Pantanowitz and colleagues^73^ reported promising results in a blinded clinical validation study after deployment of an AI-based algorithm for prostate cancer diagnosis that was developed on the basis of scanned routine slides. The AI tool was implemented in routine clinical workflow as a second-read system. The algorithm performed well and was able to distinguish Gleason grades and perineural invasion, thereby showing that AI has the potential to alleviate the diagnostic burden of pathologists.^74^ In HIC countries with existing pathology infrastructures, AI tools need to perform as well as pathologists to gain acceptability. However, in LMICs that have far fewer pathologists, the risks of relatively subtle diagnostic errors with AI-based systems might be outweighed by the benefits of the mass processing ability of AI. In such a context, the establishment of robust sample collection and processing (especially digital upload and barcoding) systems would become paramount.

We envisage an increasing role for AI in alleviating shortages of pathologists, especially in LMICs. The role of the pathologist is likely to evolve to involve establishment and overseeing of the quality assurance of widely distributed digital systems rather than the review of individual cases. This potential evolution is not unique to pathology—similar developments are likely to apply to radiology. Overall, we believe that investment in AI will bring wide-ranging benefits across the spectrum of medical services in LMICs, and should be considered a high priority.

### Facilitating diagnostic access in LMICs

The challenges faced in diagnosing prostate cancer in LMICs are likely to increase in line with the projected rising incidence of disease.^5^ Mortality due to prostate cancer has been decreasing in HICs but is increasing in LMICs, and in sub-Saharan Africa and Central America prostate cancer remains the leading cause of male cancer deaths.^23,75,76^ WHO is appropriately emphasising early diagnosis of advanced disease as a priority. Any strategies to improve cancer outcomes need to include health-care providers, local, regional, and national governments, aid networks, and charities (WHO advocates for a national cancer control plan^77^). They should also address the myriad reasons for poor prostate cancer outcomes in LMICs, including financial constraints, low levels of education, cultural and religious factors, stigma, and fear.^76,78^ Finally they should include downstream resources to facilitate appropriate treatment and management.

In LMICs, the main problems are under-detection of prostate cancer and late presentation. More than 50% of men in LMICs already have advanced disease when their prostate cancer is diagnosed, and in Africa, more than 60% of patients die from the disease.^1,2,5,10^ If the proportion diagnosed late were reduced from a half to a third, this death rate would be halved. Transrectal ultrasonographically guided biopsy is likely to remain the mainstay of diagnosis in LMICs, because in advanced disease the risk of missing a tumour is low with this approach (in HICs, cancers are more likely to be smaller at detection and thus more likely to be missed). However, it is associated with a substantial risk of sepsis, and so we recommend that the transperineal route is preferentially used. The priority in LMICs is to increase early detection, including by using PSA testing, to reduce the proportion of men presenting with metastases and increase the proportion presenting with clinically significant but curable cancer. Even for men presenting with metastatic disease, earlier diagnosis is important—it could help to reduce catastrophic presentations like spinal cord compression and urinary retention, for example.

Preliminary investigations of targeted screening has shown benefits. In a community-led PSA screening programme in a high-risk predominantly Afro-Caribbean population in the Grand Bahamas, 315 of 1844 men screened had increased PSA concentrations or abnormal digital rectal examination results, or both.^79^ On the basis of these findings, 45 men were offered a biopsy, of whom 40 had prostate cancer (2·2% prevalence in the study population), mostly high-risk disease.^79^ In a study^80^ done at a centre in São Paulo, Brazil, 9692 men underwent PSA testing and digital rectal examinations, and 588 had abnormal findings. 251 cases of prostate cancer were confirmed by biopsy (prevalence 2·6%), of which 75 (30%) were intermediate and 108 (43%) high risk. These are much higher rates of detection than associated with PSA testing in HICs, where 10% of people screened will typically be referred for further tests, such as MRI with or without biopsy, and around 1% diagnosed will be diagnosed with prostate cancer.^40,41,43^

Solutions such as pop-up clinics and mobile testing are attractive options. These approaches are used to screen for other diseases, such as HIV, in South Africa, and are cost-effective,^81^ can be led by nurses, and can be linked to education and outreach services. A pilot study, the Man Van project, which is led by one of the Commissioners (NDJ), shows the potential effectiveness of this principle for prostate cancer, albeit in a HIC. This mobile health clinic offers health checks, including PSA testing, in high-risk communities in London, UK (ie, areas of high deprivation with substantial ethnic diversity). Data from the pilot study, which included around 600 men, showed that 14 (3%) of the 422 participants who underwent PSA testing had prostate cancer.^82^ 15–20% had hypertension and pre-diabetes (and 5% had overt diabetes). Further assessments are incorporating polygenic risk score stratification into the Man Van project. Combining education with broader health tests (see Part 6) can be effective and offers a model that can be adopted both in HICs and LMICs to enable targeted screening for a range of health conditions, including prostate cancer. Additionally, the success of the Man Van Project suggests that a key component of early detection is incorporation into a broader programme of patient information and empowerment. As discussed in detail in Part 6, the broad availability of smartphones and use of social media provide potentially powerful means of improving awareness and assisting with navigation within health-care systems.

### Conclusions

In HICs, relying on opportunistic informed choice-based testing of PSA and symptomatic presentation of disease for diagnosis results in over-testing in older men and under-testing in younger men at high risk. However, too many men still present with advanced disease, especially if from socioeconomically deprived backgrounds. Targeted PSA testing focused on younger men (ie, aged 45–69 years in Black populations and 50–69 years in other populations) linked to education and outreach programmes could reduce overdiagnosis in older men and increase diagnosis in high-risk subgroups of younger men. MRI-based assessment before biopsy referral reduces over-diagnosis and overtreatment while detecting potentially lethal disease.

In LMICs, too many men present with advanced disease—a major societal problem causing suffering, early death, and financial hardship for families. Additionally, prostate cancer is just one of a range of diseases, including other cancers, cardiovascular disease, and type 2 diabetes, that are set to become substantially more prevalent in LMICs in the near future. Holistic solutions enabling early detection of all these conditions should be prioritised.

Raising awareness of prostate cancer plays a central role in effective early detection. To improve diagnosis and management of prostate cancer globally (especially in LMICs) and reduce morbidity and mortality, education is crucial and needs to adapt to novel digital approaches. We summarise our key recommendations related to prostate cancer diagnosis in panel 3.

## Part 3: Management of localised prostate cancer

Optimal management of prostate cancer requires the availability of imaging and pathology for diagnosis, surgery and radiation oncology for treatment of localised disease, radiotherapy and drug therapy for management of metastatic disease, and access to palliative therapies. Previous *Lancet* commissions have discussed restricted access in LMICs to surgery and anaesthesia,^9^ radiotherapy,^7^ diagnostics,^83^ and palliative care and pain control.^84^ Limited access to anticancer drugs was reported in a survey^85^ published in 2021. Although there could have been some improvements in the delivery of surgery and radiotherapy since the *Lancet* Commissions on these topics were published in 2015, a shortage of trained staff and facilities remains a major barrier to optimal management of patients with prostate cancer.^7–9,85^

Prostate cancer care is multidisciplinary and has changed with the development of new classes of drugs (eg, taxane chemotherapy, androgen receptor pathway-targeting agents, radiopharmaceuticals, PARP inhibitors) and new surgical, imaging, and radiotherapeutic technologies (eg, robot-assisted surgery, new generation imaging such as whole-body MRI, PSMA PET, and CT, intensity-modulated and image guided radiotherapy [which allows more precise delivery of bigger doses of radiation, while sparing dose limiting normal tissues], and stereotactic ablative radiotherapy [whereby very high doses of radiation are delivered to small volume tumours with the intention of complete obliterating the lesion]). Another key development has been the ability to dynamically shape the beam profile during radiotherapy using multi-leaf beam collimators.^86^ The combination of better imaging, the linkage of imaging to treatment delivery, and monitoring linked to advanced beam shaping has transformed the ability to deliver high-dose radiotherapy. Newer technologies, such as proton (or larger charged particle beam) therapy—ie, using protons in place of x-rays—offer alternative ways to delivery high doses with precision. However, at present these machines are large, very expensive and of limited value in prostate cancer care, and thus will not be considered further in this Commission. In the remainder of this section, we signpost the essential elements of treatment of localised prostate cancer in HICs and LMICs.

Non-metastatic disease can be managed in three ways: observation with deferred treatment according to need, surgery, or radiotherapy. Each of these options are probably available to some extent in most countries, but ease of access will differ substantially. Treatment needs will be heavily driven by local diagnostic services. Earlier diagnosis will result in a frame-shifting of the case mix to earlier stage disease—ie, fewer patients presenting with advanced disease and more presenting with early, more easily cured disease.

### Active surveillance and watchful waiting

Active surveillance, which is also known as active monitoring, comprises regular surveillance with various combinations of imaging, PSA surveillance, and clinical examination. Treatment is initiated if there is evidence of disease progression (eg, grade progression in repeat biopsy samples, disease progression on imaging, development of extra-prostatic spread) or sometimes if requested by patients (eg, in people with disease-related anxiety). Active surveillance can reduce overtreatment and help patients to avoid treatment side-effects. It is generally offered to young, otherwise-fit men with low-risk or favourable intermediate-risk disease, and implicitly assumes that the patient would be fit for curative treatment. In the landmark ProtecT trial,^6,53^ active surveillance with deferred treatment for grade or stage progression was associated with equivalent 10-year and 15-year survival outcomes to immediate treatment with surgery or radiotherapy. Updated staging data in the trial suggested that around a third of participants who were diagnosed with prostate cancer on the basis of raised PSA concentrations had intermediate or high-risk disease. At 15 years’ follow-up, 75% of men in the active surveillance group had undergone some type of further therapy. Although there were no survival differences, men who were randomly assigned to active surveillance were more likely to receive long-term androgen deprivation therapy (discussed in Part 4) than those who underwent immediate surgery or radiotherapeutic treatment, suggesting a trade-off between upfront radical treatment and later palliative treatment in some men. Patients with clinically significant prostate cancer, especially patients younger than 70 years, are at increased risk of death from prostate cancer if managed conservatively,^87^ and therefore intervention with surgery or radiotherapy is usually recommended. However, 47% of the men in ProtecT who developed metastatic disease initially had low-risk disease, suggesting that algorithms for predicting the risk of metastatic relapse are imperfect. Notably, participants were recruited to ProtecT before the widespread use of MRI before biopsies. Contemporary patients’ disease might thus be more accurately staged, leading to a potentially reduced risk of metastasis for low-grade cancers.

In HICs, many men are suitable for active surveillance and further research is needed to define the most cost-effective ways of delivering it. Low-risk prostate cancer is increasingly managed in HICs via active surveillance.^88^ Increased use in countries where active surveillance is less common is an easy win in terms of harm reduction and resource optimisation. However, active surveillance schedules are not standardised at present (in ProtecT, the schedule was largely based on PSA tests, whereas other series^89^ incorporate digital rectal examinations, regular repeat biopsies, and, more recently, repeat MRI scans), and more research is needed to define the optimal approach. Additionally, in LMICs, use of active surveillance is likely to be much less common because fewer men are diagnosed at an early enough stage to enable it.

In older, less fit, asymptomatic men with more advanced disease and those with more comorbidities an alternative form of observation is used: watchful waiting. In watchful waiting, further investigation and treatment are initiated only if new symptoms develop, with the aim of deferring the start of palliative hormone therapy as long as possible. In the SPCG-4 trial,^53^ watchful waiting was associated with reduced overall survival compared with surgery in men fit for radical therapy. Although surgery was associated with a 38% reduction in survival (hazard ratio 0·62 [95% CI 0·44–0·87]; p=0·01), the absolute difference in survival was strikingly small, with a number needed to treat to prevent a prostate cancer death of around seven in men younger than 65 years and 15 in older men (due to deaths from other causes).^90^ Delaying treatment until clinical deterioration (ie, the appearance of new cancer-related symptoms) can be safe provided men are being monitored.

Because most men in LMICs are diagnosed at a later stage than those in HICs, when few curative options tend to be available, the clinical priority is to identify those who need immediate treatment, such as those with symptoms. Even in LMICs, some people will be diagnosed with comparatively indolent disease and can be offered deferred treatment. Robust, cheap monitoring systems need to be in place to ensure that deferred treatment is safe.

### Surgery: opportunities for improvement?

Surgery has key roles in diagnosis (eg, biopsy, staging examinations), treatment (including palliative procedures such as transurethral resection of prostate and orchiectomy), and relieving urinary outflow obstruction and hydronephrosis. Radical prostatectomy is widely practised in HICs to treat localised prostate cancer. For patients with clinically significant localised prostate cancer and good life expectancy (ie, >10 years), management with curative intent using either surgical approaches or radiotherapy is recommended. The most important determinant of good outcomes (ie, long survival with minimal side-effects) after surgery is the surgeon and centre’s levels of experience.^91^ Low surgical volumes are associated with high operative mortality.^91^ Robot-assisted surgery has become popular in HICs and its outcomes are largely similar to those after open surgery.^92^ In view of the findings in ProtecT that 15-year prostate cancer-specific survival did not differ among men diagnosed after PSA testing whose initial disease was managed with surgery, radiotherapy, or initial surveillance,^85^ there is a need to better define who benefits from surgery. On the basis of available evidence, radical prostatectomy seems to offer only small increases in survival compared with more conservative non-surgical approaches, and this benefit is limited to men with long life-expectancy.^6,90^

In LMICs, where late diagnosis predominates, the priority for surgical services should be to perform biopsies for diagnosis and orchiectomy and other procedures for palliation of men with incurable disease. If early diagnosis becomes more common, LMICs will need to expand curative surgery services. Initially the surgeries offered are likely to be open or laparoscopic rather than robot-assisted procedures, which are more expensive. The timeframe for surgical training is long, so capacity expansion in LMICs needs to begin simultaneously with measures to improve early diagnosis so as not to create future capacity issues.

According to the 2015 *Lancet* Commission on surgery,^9^ 5 billion people worldwide do not have access to safe surgery and anaesthesia, and nine in ten people in low-income countries cannot access basic surgical care. Trained specialty surgeons, including urologists, are rare. The number of urologists in Africa was low, with one urologist for every 75–115 cases of prostate cancer.^9^ The ratio of urologists to population in LMICs varies from around 1:750 000 to 1:2 million.^9^ By comparison, in the UK, there is one urologist for every 50 cases of prostate cancer and for every 60 000 people.^93^ Given that prostate cancer cases and deaths are set to increase substantially in LMICs (figure 5) with little or no increase in most HICs, there is a great need to train more urologists in LMICs, whereas numbers in HICs appear appropriate.

As an example, in Nigeria, the number of urologists would need to immediately be increased from 130 to 300 for there to be one urologist for every 50 prostate cancers. However, with cases projected to increase by around 235% in the next 15 years, expansion from 130 to about 700 urologists would be required to maintain this ratio. Corresponding increases would also be required in the numbers of oncologists, pathologists, and other related specialist staff. Because undergraduate and postgraduate training in medical specialities takes 12–15 years, management of the projected rise in cases cannot be modelled exactly on care systems in HICs, and an approach using different skill mixes to facilitate delivery should be implemented to meet the immediate challenge alongside schemes to increase numbers of urological surgeons. A possible solution, which has already been implemented in some parts of Africa, is the creation of regional hubs where critical mass of expertise can be sustained, allowing development of sub-specialist services and that can provide infrastructure for training. For example, Uganda has established four regional cancer hubs with this aim.^94^ Some middle-income countries, such as India (approximately 5000 urologists; one per every eight cases of prostate cancer) and Brazil (7700 urologists; one per every ten cases of prostate cancer) might already have adequate surgical urologists, so with political will, sufficient numbers can be achieved in other middle-income countries too.

In LMICs with a shortage of trained urologists, biopsies might not be needed to establish a diagnosis of prostate cancer in men with raised PSA and typical disease presentation, and simple surgical procedures, such as orchiectomy, could potentially be done by trained clinician assistants (in a previous trial,^95^ hernias repaired by clinician assistants had similar outcomes to those repaired by surgeons). Many men with prostate cancer who are unlikely to be able to afford medical treatment with gonadotropin-releasing hormone (GnRH) agonists could benefit from orchiectomy, and training clinician assistants to undertake this cost-effective, easy-to-perform intervention offers substantial potential to improve outcomes. In view of the psychological impact of orchiectomy, as evidenced in men treated for testicular cancer,^96^ there has been a move to less mutilating techniques, such as subcapsular surgical approaches, which are associated with less severe psychological effects.^97^ Because the endocrine effects are broadly similar whether pharmacological or surgical approaches are used (and could even be less severe with orchiectomy^98^), a policy of offering surgery after initial stabilisation on GnRH agonists, when both the financial and medical effects of treatment are apparent, might be more acceptable than offering surgery as primary therapy, although this approach is dependent on the availability of resources. Sub-capsular orchiectomy can be offered via clinician assistants. Overall, prescribing orchiectomy rather than GnRH agonists could allow resources (both direct cost savings and staff time) to be diverted to alternative treatments that confer additional survival benefits or reduce complications, such as low-dose abiraterone or upfront docetaxel. In Brazil, for example, orchiectomy costs around US$85, whereas GnRH analogues cost $59 monthly. Given that around 85% of men in Brazil take GnRH analogues, substantial saving could be made by performing orchiectomies instead, with no deleterious effects on overall survival.

In summary, radical prostatectomy cannot be recommended as a high treatment priority in most LMICs in the short term. However, in the mid-to-long term, if more resources are devoted to detection and diagnosis of prostate cancer, a stage shift will occur, with a resulting increase in demand for surgery (and radiotherapy). In planning early diagnosis services, LMICs will need to urgently implement the expansion of surgical services to meet the future case load, which will have substantial additional benefits beyond the care of men with prostate cancer.

### The role of radiotherapy: gaps and opportunities

Access to radiotherapy is required for optimal management of almost all cancers. For prostate cancer, radiotherapy is a key component of both radical and palliative care. It has a potentially curative role in non-metastatic disease and an important life-extending role in low-burden metastatic disease. In general, prostate cancer outcomes are improved by combining radio-therapy with androgen deprivation therapy in a risk-stratified fashion—ie, high-risk disease is treated with a longer duration of androgen deprivation therapy than low-risk disease. Radiotherapy also offers great benefit in locally advanced and locoregional disease (as well as in palliation of metastatic disease) by relieving pain and alleviating complications such as spinal cord compression,^99^ and there is increasing evidence for its role in the management of oligometastatic disease.^100–102^

**Figure 6:**
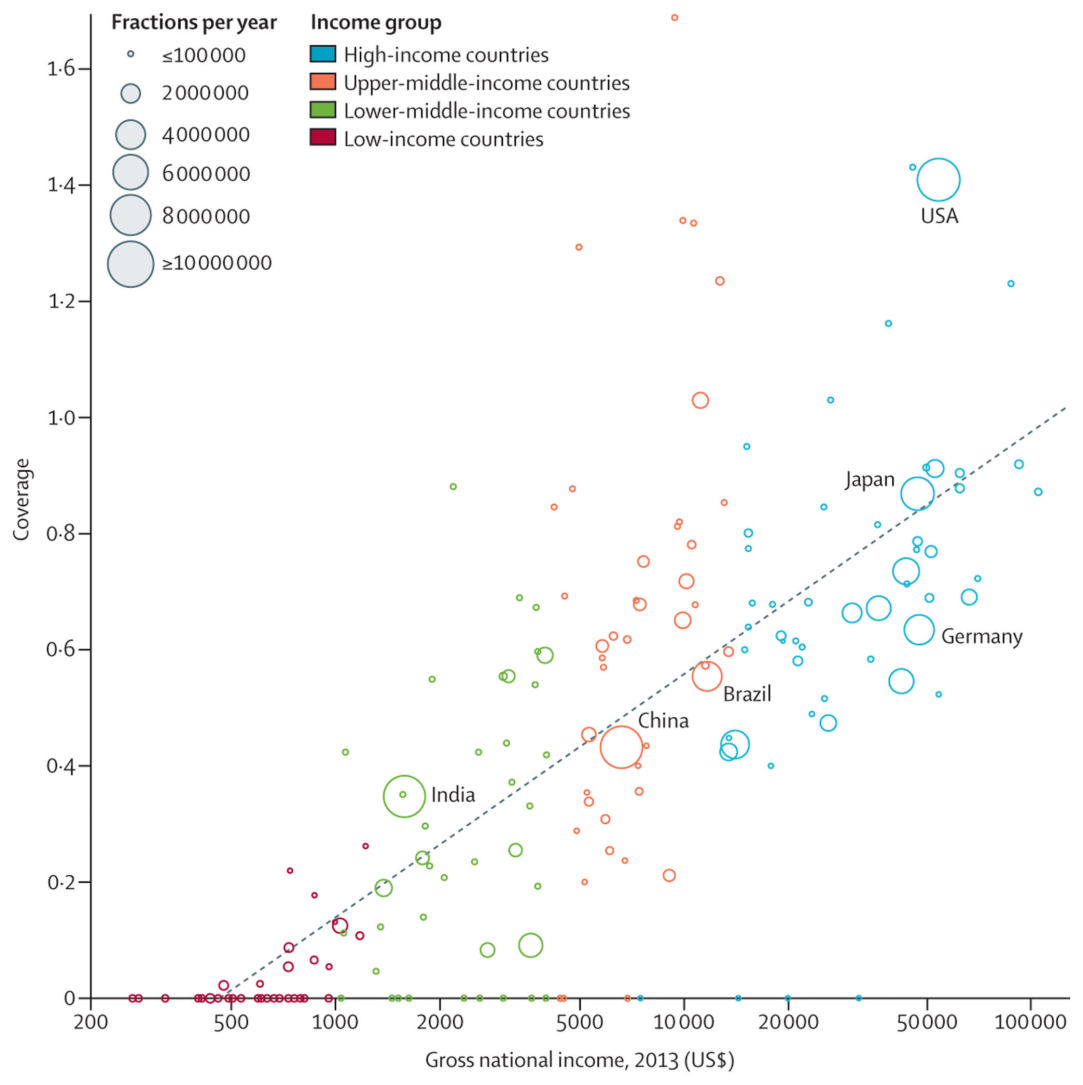
Radiotherapy coverage as a function of gross national income Each circle represents a distinct country. The diameter of the circle is the actual yearly number of fractions delivered. Coverage is reported for an assumed 8 h operating day. Source: Atun et al (2015).^7^

**Figure 7:**
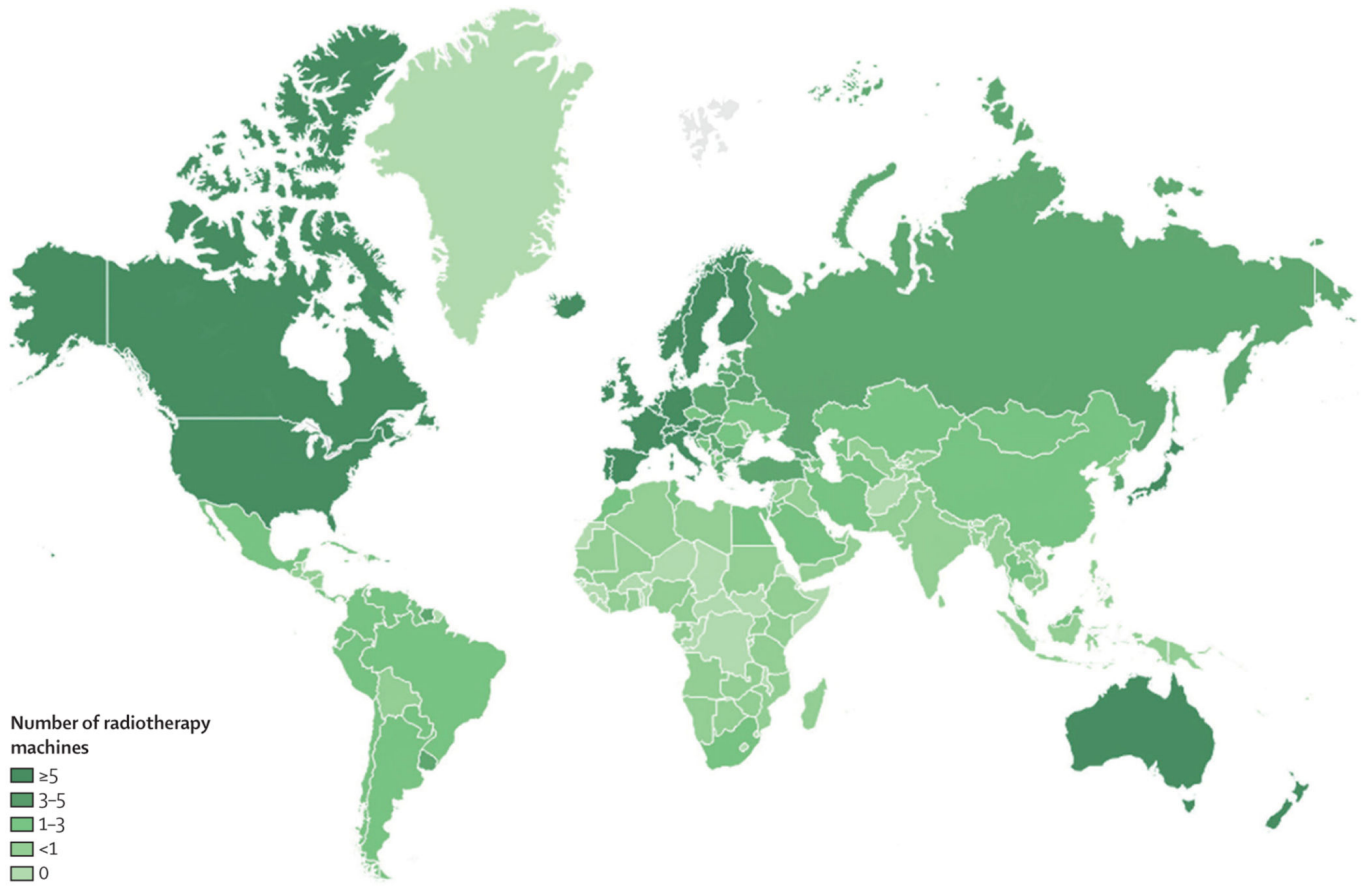
Access to radiotherapy worldwide per million population Source: Abdel-Wahab et al (2021).^104^

However, the 2015 *Lancet* Commission on radiotherapy^7^ showed that worldwide access to radiotherapy is variable, with up to 90% of patients in low-income countries having no access. Access was roughly proportional to gross national income (figure 6). Data^103^ from the Directory of Radiotherapy Centres show that, as of 2020, North America and western Europe had 6–11 radiotherapy machines per million people, whereas in Africa there were 0–1·7 machines per million population and in southeast Asia there were 0·5 machines per million population (figure 7). Several low-income countries had no access to radiotherapy.^8,103^ Investment in radiotherapy in LMICs would save many lives and would be cost-effective.^7,105^ Programmes such as the International Atomic Agency’s Rays of Hope initiative,^106–108^ in collaboration with WHO, are seeking to address these imbalances by increasing provision. In panel 4, we look at case studies of access to radiotherapy in Brazil, India, and Africa, which highlight both the problems and potential solutions.

Driven by improved delivery technologies and a series of global trials^115^ comparing radiotherapeutic fractionation techniques, radical schedules have shortened from 35–40 fractions delivered over 7·5–8 weeks to around 20 fractions over 4 weeks, with a likelihood based on recent data that the number will settle at 2–5 fractions delivered over 1–1·5 weeks.^116^ These hypofractionated schedules offer clear cost savings to payers and are much more convenient for patients: the short delivery times mean disruption to home or work is minimised and travel for therapy hugely reduced, which in turn reduces geographical barriers to access. There is thus an opportunity to greatly extend access to radiotherapy by delivering hypofractionated schedules combined with androgen deprivation therapy. Palliative schedules can also be delivered very rapidly—one fraction is sufficient for most indications.^99^ We predict that the ability of AI to transfer diagnostic images, auto-contour targets of interest (ie, the tumour), critically limit dosing of healthy tissues, and auto-generate radiotherapy plans will grow rapidly, which means that much of the infrastructure associated with radiotherapy delivery could be outsourced and should become cheaper. Distributed delivery of radiotherapy, both radical and palliative, with centralised control has the potential to accelerate access in LMICs. As with surgery, the creation of regional hub facilities for radiotherapy offers a delivery model that allows the building of expertise. Combining such hubs with surgical hubs also clearly makes sense, not just for prostate cancer but across multiple disease areas.

To improve access to radiotherapy equipment and training of personnel are needed, although we recognise that LMICs are diverse and that solutions will depend on economic and political will. Partnerships between global and local enterprises should be encouraged: the low costs of local manufacturing and potential access to an underserved, growing health-care sector can attract global market leaders, provided there is governmental support. Ensuring that countries waive taxes on imported radiotherapy units would facilitate the setting up of new radiotherapy services.

Generally, radiotherapy services for poorer people within LMICs have lagged behind those provided in private cancer centres in both quantity and quality. Many public health-care centres cannot support equipment maintenance and infrastructure to run modern linear accelerators to deliver. Innovative Technologies Towards Building Affordable and Equitable Global Radiotherapy Capacity is a project funded by the UK Science and Technology Facilities Council working with 22 African countries that aims to develop new radiotherapy technologies and machines designed specifically for use in sub-Saharan Africa.^117^ Public–private partnership models could also enable private sector radiotherapy machines to be used to treat public patients during quiet periods. Additionally, cooperative training programmes could be developed for radiation oncologists, medical physicists, and radiotherapy technologists between public academic institutes and private radiotherapy centres to increase exposure to modern radiotherapy techniques.

Global collaboration is needed to train, mentor, and sustain local expertise in LMICs. The International Atomic Energy Agency has implemented several programmes to improve radiotherapy access and quality, including advisory missions of experts, audits of radiation facilities to achieve maximum use, training workshops, and staff fellowships.^118^ Organisations such as Education of Health Professionals for the 21st Century have advocated for global instructional reforms, adapted to local conditions and resources, and promote education among different LMICs.^114^ The International Cancer Experts Corps is running a project to link teams in twinning programmes or mentoring schemes.^117^ Many other global and regional initiatives are also attempting to improve access to radiotherapy, leading sometimes to duplication and wasted resources, and the International Atomic Energy Agency has recommended a central framework to coordinate these efforts.^104^

### Conclusions

The incidence of prostate and other cancers in LMICs will continue to rise in the coming decades in line with projected demographic changes, with a corresponding increasing need for radiotherapeutic and surgical facilities and expertise. International organisations, and commissions such as this one, need to lobby governments to view both surgery and radiotherapy as priorities in cancer treatment. Governments health coverage should include radiotherapy and surgery so that they are affordable to the whole population. Governments also need to fund radiotherapy units and ensure that they are sustainable with help from organisations like the International Atomic Energy Agency. Each country should invest in at least one comprehensive cancer centre that offers radiotherapy and surgery, and should require that centre to train local experts. Alternatively, regional hubs offer a possible bridge between no provision to full national services. The prevalence of prostate and other cancers in LMICs will continue to rise as life expectancy increases, with increasing need for radiotherapy and surgical facilities and expertise.

In HICs, for non-metastatic disease, the emphasis should be on reducing overtreatment in low-risk disease and reducing the treatment burden in higher-risk disease (eg, moving to hypofractionated radiotherapy schedules, avoiding excess use of androgen deprivation therapy). For advanced disease in both HICs and LMICs, core therapies should be defined and funded to maximise survival and minimise health-care costs.

## Part 4: Systemic therapy for advanced disease

Many patients in HICs and most patients in LMICs present initially or subsequently with incurable locally advanced or metastatic prostate cancer. In such patients, androgen deprivation therapy either with GnRH agonists or orchidectomy, is the standard initial treatment and provides effective palliation of symptoms in most men. At present, around 60% of men in the USA and UK receive androgen deprivation therapy only and can expect median survival of 3·5 years.^119^ Making intensified androgen deprivation therapy—ie, adding a modern oral anti-androgen drug, such as abiraterone or enzalutamide—available to men in HICs with newly diagnosed advanced prostate cancer would increase survival to more than 5 years.^120–128^

Although most men in HICs opt for medical castration with GnRH agonists, these drugs are not more effective than orchiectomy, which is cheaper and more convenient (ie, a one-off procedure rather than semi-regular injections) and should be made available as a treatment option for everyone with advanced prostate cancer. In this section, we will focus predominantly on access to therapy in LMICs, but similar issues could arise in HICs in future, where the increasing costs of new drugs is challenging for health-care providers in even the wealthiest economies. The essential treatment configurations for LMICs and HICs are summarised in the table. Median overall survival after a diagnosis of metastatic prostate cancer in HICs has improved from about 2·5 years to about 5 years since the advent of additional hormonal agents (abiraterone, enzalutamide, and others), chemotherapy (docetaxel and cabazitaxel), and radium-223, with parallel improvements in symptom control and quality of life,^120–128^ and more recent developments such as targeted radioligand therapy and PARP inhibitors are likely to produce further gains. Upfront use of many of these agents yields additional benefits (both prolongation of survival and reduction of disease complications).^120–128^ However, with the exception of docetaxel and generic abiraterone, these treatments are expensive, which limits access mainly in LMICs. The provision of access to effective new drugs at affordable prices is a major challenge, and diverting cost savings from performing orchiectomies rather than prescribing GnRH agonists to other drugs is likely to be clinically and economically effective. In panel 5, we review the availability of prostate cancer drugs in Brazil, India, and some African countries to illustrate the broader issues in LMICs, and consider barriers to access and strategies whereby people might access effective drugs.

### Pharmacoeconomics: a way to improve access to drugs?

High drug costs are a major driving factor for disparities in prostate cancer outcomes. A WHO report^136^ on cancer drug pricing stated that “Pharmaceutical companies set prices according to their commercial goals, with a focus on extracting the maximum amount that a buyer is willing to pay for a medicine.” As a result, cancer drugs are often unaffordable. The report found little evidence of a link between the costs of research and development and the price charged for the final product, with the authors noting that returns on investment for cancer drugs are high and pricing policies are often opaque. The report also stated that “the rate of growth of expenditure on cancer medicines greatly exceeds the rate of growth of newly diagnosed cancer cases…the growing expenditure may be primarily due to increases in medicine prices or a shift towards using higher-cost cancer medicines. In addition, the rate of growth of expenditure on cancer medicines exceeds the rate of growth of overall health care expenditure.”^136^

The result is that large swathes of the global population cannot access the most effective cancer therapy. Many effective anticancer drugs were developed because of publicly funded research and we believe that it is unethical that the profit motive prevents such drugs from being available everyone who could benefit from them, particularly people in poor countries. The roll out of HIV medication^137^ and more recently COVID-19 vaccines^138^ show that it is possible to combine profitable drug development with the broader public good. Non-availability of life-prolonging treatments is not always due to the cost of manufacture. Even in HICs, many men receive suboptimal prostate cancer therapy, because of the need to pay for therapy out of pocket (rather than having it covered by insurance or the state).^139^ For example, some expensive androgen receptor-targeted therapies are not currently funded by the UK’s National Institute for Health and Care Excellence. Risk factors that increase the likelihood of having to pay out of pocket for treatment include young age, low household income, non-White race, being unmarried, marital status, geographical location, and comorbidities.^140^ Many more patients have little access to optimal treatment in LMICs.

In 2019, Ratain and colleagues^141^ introduced the concept of interventional pharmacoeconomics, with the aim of decreasing costs of therapy through development of new dosing regimens while maintaining equivalent efficacy. There are four possible strategies of interventional pharmacoeconomics: dose reductions, less frequent doses, reduced treatment duration, and therapeutic substitution. For treatment of prostate cancer, pharmacokinetic and pharmodynamic studies^142,143^ have shown that the dose of abiraterone can be reduced without compromising efficacy. 250 mg abiraterone taken with breakfast had similar effects on target adrenal androgen and on PSA concentrations as the recommended dose of 1000 mg after an overnight fast—a potential cost reduction of 75%.^144^ The National Comprehensive Cancer Network now recommends 250 mg abiraterone with food as an alternative dose, adoption of which led to large savings in India.^144^ This dose can be recommended whenever abiraterone is prescribed in LMICs. Enzalutamide, an alternative oral anti-androgen on WHO’s list of essential medicines can also be given at lower than recommended doses.^145^

Few trials have assessed the duration of androgen deprivation therapy prescribed, although intermittent hormonal therapy appeared equivalent to continuous treatment in randomised controlled trials^146,147^ done before the development of more recent life-prolonging therapies. The role of intermittent androgen deprivation therapy is less certain since the benefits of adding treatment with drugs such as abiraterone or enzalutamide to continuous therapy have been shown for men with hormone-sensitive prostate cancer.^121,125,128^ However, if abiraterone or enzalutamide are not available, intermittent androgen deprivation therapy is a sensible option for men in LMICs who do not opt for orchiectomy and could reduce both side-effects and cost. Intermittent androgen deprivation therapy might also be appropriate for men with low volume metachronous relapses^148^ or so-called PSA relapses (ie, relapses detected solely by a rise in PSA) after primary therapy, in whom prognosis is good and risk of side-effects from overtreatment are high.^146,147^ The optimal duration of hormonal therapy in men who remain in remission is unresolved. For example, the benefit of 2 years of abiraterone (with or without enzalutamide) in the STAMPEDE study^149^ of men with high-risk non-metastatic disease seemed similar to that achieved with longer durations of abiraterone in metastatic disease. There is thus a need to explore not only the minimum dose needed for benefit but also the minimum treatment duration: using too much of a drug for too long can have harmful clinical as well as financial effects. Because pharmaceutical companies are unlikely to fund studies of reduced dose regimens or shorter regimens, key stakeholders such as governments and health-care systems should be encouraged to do so. The potential savings in drug costs from dose reduction could easily cover the costs of the trial. Single payer systems, such as the UK’s NHS, offer a cost-effective route to a wide range of trials.

Near-equivalence studies that combine various types of evidence to support the acceptability of an alternative treatment relative to standard of care have been proposed.^150^ Such studies often challenge the commonly held belief that more treatment is better. Near-equivalence studies provide an alternative approach to classical non-inferiority studies, which require large sample sizes and in which toxicity, quality-of-life, and cost are often only secondary endpoints. Under the principle of near equivalence, an alternative drug in the same class as approved standards of care could potentially be used even if not specifically assessed for the same type and stage of cancer, drugs that failed in non-inferiority studies, which sometimes show similar outcomes even though they do not meet the predefined statistical threshold, could be reassessed, and evidence of pharmacodynamic and pharmacokinetic efficacy could be combined with data from small clinical trials to support use. The 72-patient randomised controlled dose-comparison study of abiraterone^143^ that established that a substantially reduced dose of the drug could potentially be as efficacious as the standard dose is an example of a near-equivalence trial. In such trials, financial toxicity should be treated with the same emphasis as physical toxicity.

### Generic drugs

Generic drugs are prescribed widely and represent the most common form of therapeutic substitution. India is the largest global producer of generic medicines (including those for treatment of prostate cancer), with more than 3000 companies and 10 500 manufacturing facilities, and exports these generics to the USA and various countries in Europe and Africa, among others.^151^ Generics cost less than the original drug—sometimes as much as 90% less, depending on where they are marketed.

Research supports the safety of generic drugs but results vary with regard to equivalence to brand-name drugs. For example, in a study^152^ in which the pharmaceutical quality of 31 generic docetaxels was compared with that of the brand name Taxotere (produced by Sanofi-Aventis), only three of the ten Indian brands assayed included more than 90% of the expected mass of docetaxel. In an analysis^153^ of 3·5 million patients in two US commercial insurance databases (Optum Clinformatics Data Mart, 2004–13, and Truven MarketScan, 2003–15), generic drugs for treating common chronic illnesses were associated with similar clinical outcomes to brand-name drugs. However, many physicians, pharmacists, and patients viewed the generics as inferior because of real or perceived inferior quality and regulation of drugs (especially generics produced in LMICs). Pharmaceutical companies in LMICs continue to strengthen their regulatory and manufacturing processes, with improved automation, operating procedures, and quality management. In the meantime, we propose a method for selecting high-quality generics involves three broad steps: selection of the generic manufacturer based on criteria that assess the robustness of the company, a technical assessment of properties of the generic drug, and financial analysis of the top two or three generics identified by the first two steps before inclusion in the hospital pharmacy. Various national bodies, such as the US Food and Drug Administration, have robust licensing processes for generic medicines, which function as a reference source.^154^

The availability of generics for prostate cancer has led to substantial improvement in access to life-prolonging drugs in LMICs. For example, since the 2018 expiry of the patent on abiraterone, at least 15 generic brands have become available in LMICs at a cost of $100–200 per month for full-dose treatment ($25–50 per month if the 250 mg per day dose is prescribed), more than 90% cheaper than the brand name version.^144^ Similarly, generic enzalutamide and cabazitaxel are available in LMICs at a fraction of the cost ($300 per month and $150 per cycle, respectively) of the brand-name versions.^129^

### Opioid analgesics and supportive care

**Figure 8:**
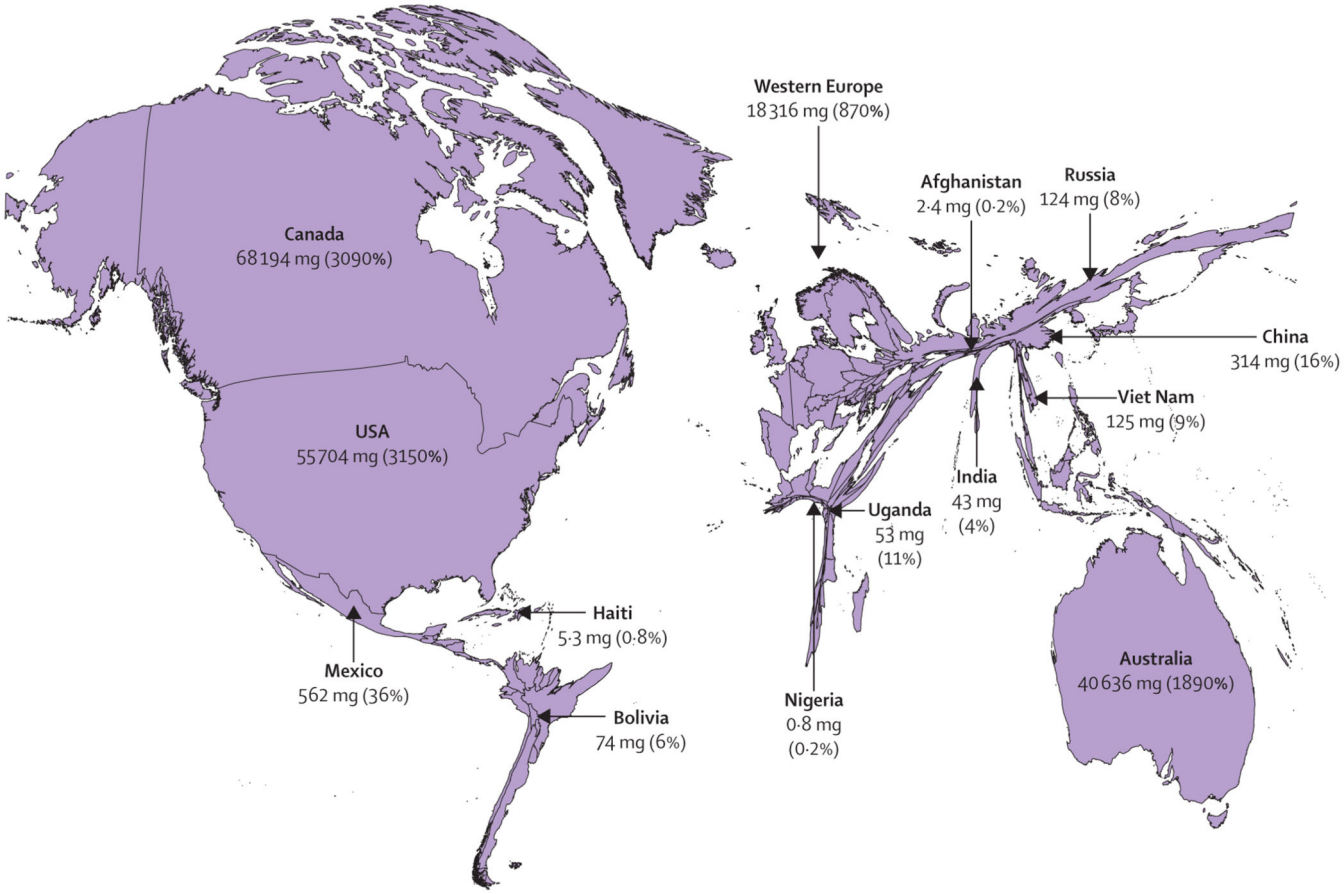
Distributed opioid morphine-equivalent (morphine in mg per patient in need of palliative care, average 2010–13), and estimated proportion of need that is met for the health conditions most associated with serious health-related suffering Source: International Narcotics Control Board and WHO Global Health Estimates, 2015. Reproduced from Knaul et al (2018).^84^

Prostate cancer is associated with a high frequency of bone metastasis.^4^ Late-stage refractory disease is thus often characterised by bone pain, and access to good pain relief, especially where access to palliative radiotherapy is limited, is key for good palliation. The *Lancet* Commission on palliative care and pain relief^84^ reviewed problems with access to pain relief in LMICs. The most relevant issue identified for prostate cancer was poor availability of opioid analgesics. This issue was also highlighted in the *Lancet* Commission on cancer in sub-Saharan Africa.^134^ Opioid use varies substantially worldwide (figure 8). Although in some countries, over-use of opioids has resulted in addiction problems, low availability of opioid analgesics in many LMICs causes major suffering, including for people with prostate cancer. Often this shortage is not due to cost constraints, but rather to regulations that restrict availability with the goal of decreasing addiction and drug trafficking.^134^

In 1985, the Indian Government adopted stringent legislation to regulate narcotic misuse and trafficking. As a result, medical use of morphine decreased by 97%, which severely limits access for pain management.^155^ Harsh regulations, a complex licensing system, and reluctance to prescribe and use opioids among health-care providers and the public were barriers to opioid access.^156^ Although opioids were included in hospital formularies, availability was severely restricted because hospitals found the regulatory requirements difficult to meet, and there was impetus for regulatory reform from clinicians involved in palliative care.^157^ Bureaucratic hurdles, insufficient training in pain management and opioid use, and poor understanding of the benefits and adverse effects continue to be a challenge for opioid access and use in India. An amendment to the Narcotic Drugs and Psychotropic Substance Act in 2014 improved opioid access, but there is a lag in implementation across the country.^158^ Similar barriers were found to greater or lesser extents across southeast Asia.^158^

In most African countries, pain management follows the WHO pain ladder, with both non-opioids and opioids prescribed.^159^ The Treat the Pain programme,^160^ an initiative of the American Cancer Society, has provided access to morphine in partner countries in sub-Saharan Africa. Some pharmaceutical companies also have special licences to import controlled drugs. We strongly advocate for access to opioid analgesics for all men with metastatic prostate cancer who require pain relief. Attempts to prevent addiction should not restrict opioid availability to patients.

### Conclusions

In HICs, some men still initially present with metastatic prostate cancer, and many receive suboptimal therapy, even when optimal therapy is funded. A high priority in HICs is thus to define optimal therapy and put systems in place to improve access. This action is partly about the education of health professionals, but will also involve empowerment of patients (see Part 6).

In LMICs, most men present with late-stage incurable prostate cancer. For those men, early diagnosis and initiation of standard hormone therapies could reduce morbidity and prevent serious complications like spinal cord compression and urinary retention. Furthermore, use of orchiectomy rather than injected androgen deprivation therapy could free up valuable resources for more effective drug therapy, such as new generation anti-androgens. As in HICs, the introduction of these approaches also needs to be linked to education of both health professionals and the general public.

## Part 5: New technologies: personalised drug selection and novel imaging approaches

### Targeted molecular therapies

Growing knowledge about the genomic landscape of prostate cancer brings the possibility of molecular stratification of therapies and development of molecularly targeted therapies. The most promising targets have been *PSMA* and the DNA damage repair genes, particularly *BRCA2*, but also *BRCA1, ATM*, and *CHEK2*. The resultant genomic instability (due to the inability to adequately repair particular types of DNA damage) results in cancer cells having increased vulnerability to PARP inhibitors, which target one of the key DNA repair pathways that are abnormal in tumours with mutations in the genes listed. Non-cancerous cells not carrying the mutation retain the ability to repair genomic damage via alternative pathways. Olaparib was the first PARP inhibitor to be approved by the US Food and Drug Administration,^161^ and various others are being assessed in clinical trials with positive results, including talazoparib^162^ and niraparib.^163^ Various trials are underway assessing these drugs in newly diagnosed patients (eg, TALAPRO-3 [NCT04821622], a trial of talazoparib, and STAMPEDE2 [ISRCTN66357938], a trial of niraparib). Other potential targets in prostate cancer that are being assessed in trials include the PI3K–AKT pathway, which is dysregulated in cancers with functional PTEN loss and which can be targeted with drugs such as ipatasertib,^164^ immunotherapy for the small proportion of patients harbouring *MMR* mutations, and ATR inhibitors in *ATM* mutant tumours (eg, the Planette trial [NCT04564027] of ceralasertib in solid tumours with the relevant mutations). There will doubtless be further examples of targets amenable to drug therapy and new agents licensed to treat them. The challenge is to identify targets, and then, once they are found, to identify the patients who might benefit from treatment directed at this target. Because only around 10% of men with advanced prostate cancer harbour mutations in DNA damage repair genes that might benefit from PARP inhibition, upfront genomic screening needs to be factored into treatment costs. The reduced numbers of patients who might benefit from targeted molecular therapies make the trials highly complex and expensive, and necessitate novel approaches both by those who design and oversee trials and by regulators. The precedent of the fast-tracked COVID-19 vaccine trials should be examined to establish whether it could be applied in other contexts, including the development of molecular therapies for prostate cancer.

In parallel with the ability to target DNA mutations in tumours, use of ctDNA as a diagnostic tool for treatment monitoring is expanding. In early disease, ctDNA concentrations in the blood are too low to be detected by available technology.^165^ In more advanced disease, however, ctDNA is measurable and is prognostic: changes in concentrations in response to treatment could provide an early indication of outcomes.^166^ When ctDNA can be detected, it reflects tumour volume, and allows for characterisation of tumour mutations and tracking of cancer evolution.^167^ Thus, ctDNA measurement could be used as a monitoring tool and decision aid. Potentially more sensitive than imaging, ctDNA measurement is likely to feature increasingly in trials of treatments for late-stage prostate cancer.

At present, ctDNA measurement and targeted molecular therapies are at the limit of affordability in HICs. Ways need to be found to enable use of these technologies at prices that are affordable. Again, the rapid dissemination and rollout of widespread cheap PCR testing and monitoring for COVID-19 provides a precedent.

### Next-generation imaging for disease staging

**Figure 9:**
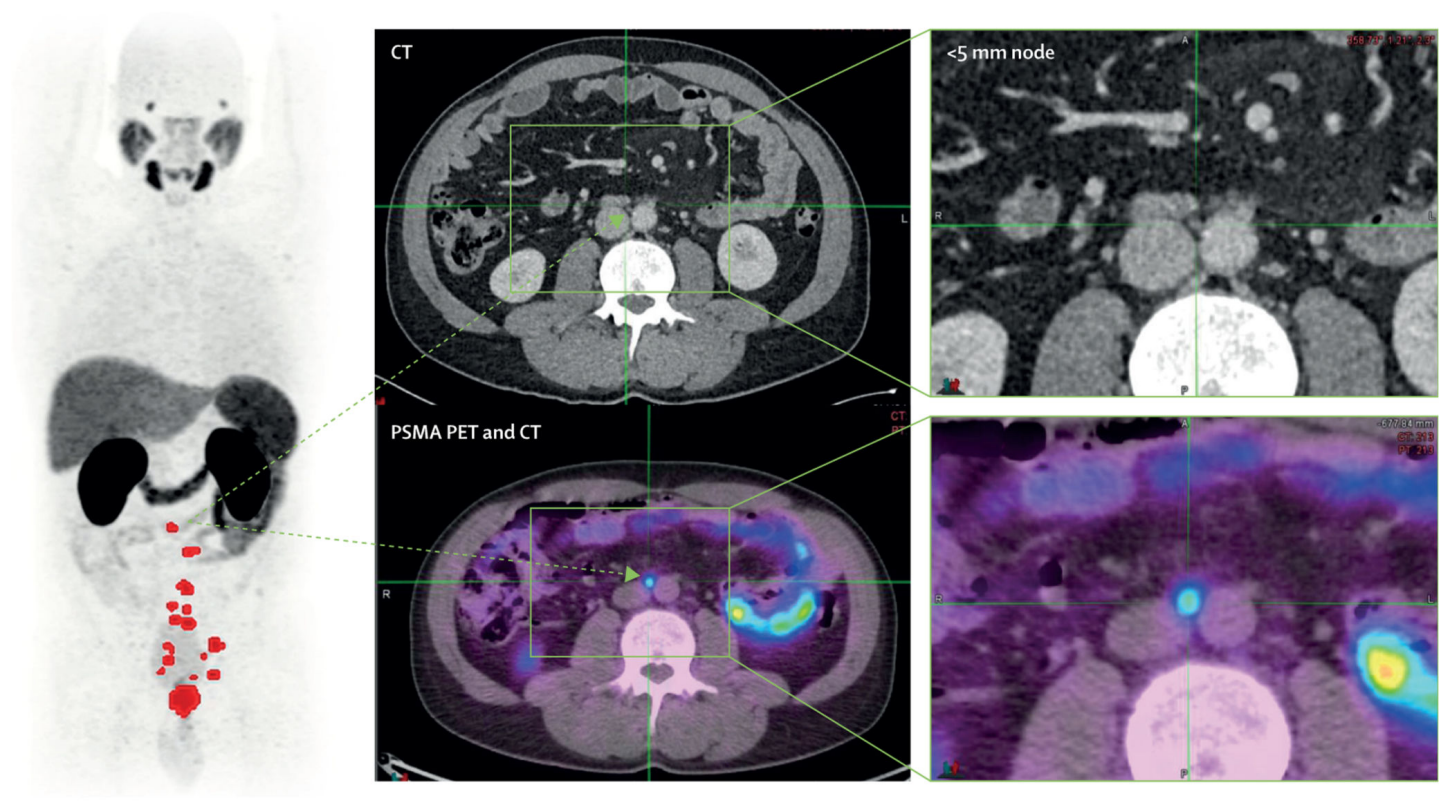
Sensitivity of PSMA PET and CT compared to CT alone in metastasis detection The left-hand panel is a coronal whole-body PET image. Black areas show normal tissue tracer uptake (by the salivary glands, liver, spleen, and kidneys). The red areas represent cancer-related PET tracer uptake in metastatic lymph nodes and the prostate. The upper panels are CT images of lymph nodes with cancer in the para-aortic region. In the lower panels, the addtion of PET to CT clearly shows tracer uptake by low-volume lymph node metastasis, which would be impossible to discriminate in the CT only images. PSMA=prostate-specific membrane antigen.

Rapid changes in imaging and theranostics (in which a diagnostic targeting molecule is linked to a cytotoxic effector, usually a radionuclide, that can be delivered at therapeutic doses) have been reviewed in a 2021 *Lancet Oncology* Commission.^168^ Here, we highlight issues of relevance to prostate cancer. So-called conventional methods for detection of metastases include radiography, CT, MRI, and radionuclide bone scintigraphy. These methods have low sensitivity for detection of nodal and early bone disease, which can lead to variations in outcomes among prognostic groups, since these results affect therapeutic choices. For example, in the ProPSMA study,^169^ men with prostate cancer were randomly assigned to undergo either conventional or PET-based disease assessment. Management changes occurred in 27% of participants when PET-based assessment was added to conventional imaging compared with 7% of participants when conventional imaging was added to PET-based assessments.^169^ Figure 9 shows how the addition of PET to CT allows detection of low-volume disease in soft tissue structures in particular (without PET, the grey-scale CT images do not allow tumour and, for example, nodal tissue to be distinguished). Higher-sensitivity next-generation imaging, such as PET and whole-body MRI, are increasingly used for staging patients, because the presence or absence of metastases affects decision making. For PET, a range of radiopharmaceuticals are used, each with advantages and disadvantages.^170^ Evidence suggests that next-generation imaging provides more accurate staging of intermediate-to-high risk prostate cancer and can detect metastatic sites in men with biochemical recurrence (ie, rising PSA concentrations) in cases in which no metastases were apparent on conventional imaging.^169^ Standardised reporting guidelines have been developed by the European Association of Nuclear Medicine,^171^ as have appropriate use criteria in both non-metastatic high-risk and recurrent disease settings.^168,172^ Next-generation imaging has also been incorporated into clinical guidelines, such as those of the National Comprehensive Cancer Network,^130^ but evidence for the effect of such imaging on long-term prognosis or outcomes such as survival is scarce.

There are concerns about the use of next-generation imaging to detect occult metastasis,^173^ given that it will lead inevitably to stage migration (ie, whereby patients with similar disease are put in different categories because of the use of different staging techniques—the so-called Will Rogers phenomenon^174,175^), which makes application of data from previous trials difficult and can lead to artifactual improvements in outcome because the worst patients in better prognosis groups will be shifted to worse prognosis groups. The effect is that the better group now records improved average outcomes (as the worst patients have been removed) and, paradoxically, the worse prognosis group now also reports better outcomes (because the new patients added have a better prognosis than those who were previously in the group). Overall, however, the two groups together are unchanged and hence the apparent improved outcomes are spurious. Also, adoption of next-generation imaging will not improve overall cancer-specific survival in the absence of therapies such as stereotactic ablative radiotherapy^101^ that can be more accurately targeted as a result of the improved imaging capabilities. Novel trial designs that show clinically meaningful benefits (eg, from treatment of oligometastases) are needed before general adoption of next-generation imaging detection can be recommended for general adoption into clinical practice.^176^

Assessment of the efficacy of treatments used in the management of metastatic disease is important in routine clinical practice. For soft tissue disease, assessment is straightforward: volume changes of known disease sites can be assessed with routine CT.^177^ By contrast, measurement of progress in treating bone disease is challenging: it is based on the destructive appearance of the bone that surrounds disease residing within the marrow space.^178^ Imaging becomes increasingly important in monitoring therapy in late-stage prostate cancer, because of the unreliability of markers such as PSA concentrations.^179^ Conventional and next-generation imaging can be prognostic, with visceral involvement and lytic bone disease associated with poor prognosis.^119,180^

Increased use of PET-based functional imaging will also lead to stage migration. In the STAMPEDE trial,^181^ use of prostate radiotherapy, in addition to standard systemic therapy, in newly diagnosed patients was associated with prolonged overall survival in men with between one and three metastases as measured by CT and bone scans (compared with receiving systemic therapy only). However, given the increased sensistivity of PSMA PET (figure 9), many men staged as having oligometastatic disease on CT scans will be upstaged to polymetastatic disease with the addition of PET. The ProPSMA study, showed that many men with high-risk M0 disease are restaged as having low-volume M1 disease when PSMA PET is used.^169^ In another study of patients undergoing surgery, the extent of stage migration (close to 11%) might have been less in patients with intermediate-high and high-risk disease when radiological stage was compared with pathological stage after surgery.^182^ At present, most imaging studies focus on sensitivity and specificity or effects on clinical decisions (which can, of course, be flawed), but increased accuracy alone does not lead to improved outcomes if it drives inappropriate treatment choices. Studies are needed to assess the effect of changes in imaging methods on clinical outcomes, like relapse-free or overall survival.

### PSMA theranostics

The same strategies that have allowed the development of PSMA-based imaging can also be used to generate therapeutic radiopharmaceuticals.^183^ Radionuclides used for diagnostics, such as gallium-68 or fluoride-18, are replaced with therapeutic radionuclides—eg, lutetium-177 (^177^Lu). In some countries, compassionate access laws enable physicians to administer unapproved drugs to patients in whom all conventional treatment options have been unsuccessful. These laws have enabled initial adoption of PSMA-targeted ^177^Lu (^177^Lu-PSMA) in Germany, for example. At the 2019 Advanced Prostate Cancer Consensus Conference, more than 50% of panel experts considered referral of patients with no other treatment options for ^177^Lu-PSMA to be appropriate.^184^ Since then, two randomised trials, TheraP^185,186^ and VISION,^187^ have suggested that ^177^Lu-PSMA is efficacious and safe in men whose disease has progressed after treatment with enzalutamide or abiraterone and docetaxel. In TheraP,^185,186^ ^177^Lu-PSMA was associated with a higher proportion of complete and partial responses, fewer toxic effects, and better patient-reported outcomes than cabazitaxel, whereas in VISION,^187^ the addition of ^177^Lu-PSMA to standard care was associated with increased overall survival compared with protocol-defined standard-of-care. In both studies, PSMA PET and CT were used to select eligible patients, because uptake of tracers is predictive of the effectiveness of anti-PSMA radioligand therapy,^187,188^ and patients with PSMA-negative disease were excluded. Although both of these studies have limitations, ^177^Lu-PSMA seems both active and well tolerated. Thus, PSMA theranostics are likely to become a new option for men with castration-resistant metastatic disease. Multiple clinical trials are underway to assess use of PSMA theranostics for earlier-stage prostate cancer.

### Challenges for clinical trials

Clinical trials are the key engine of change in clinical care, but the design of trials for men with prostate cancer is difficult for various reasons: because survival has improved, there is a need for surrogate intermediate endpoints that predict long-term survival; bone-predominant disease cannot be easily assessed in trials of treatment response (because standard bone scintigraphy is based on bone’s reaction to damage and is not a direct measure of disease volume); and the increasing use of next-generation imaging, particularly PSMA PET, is blurring the boundaries between locally advanced and metastatic disease, further complicating assessment of disease response and progression and interpretation of older trials in which conventional imaging was used. Some data suggest that metastasis-free survival is strongly correlated with overall survival in locally advanced disease,^188^ which could enable earlier publication of results from trials in advanced disease. This finding was, however, based on CT and bone scintigraphy rather than next-generation imaging. Surrogacy needs to be established in a setting-specific context and to relate to clinically relevant criteria, such as quality of life or overall survival.^189^

There is also a need to address the limitations of the Prostate Cancer Working Group 3 imaging criteria,^177^ including the absence of criteria for response in the more than 60% of patients with no soft tissue measurable disease, criteria for early progression (apparent progression detected in bone scan needs to be supported by confirmatory evidence at 6–12 weeks per the criteria), and criteria for detectable radiological progression in the at least 25% of patients who discontinue treatment on the basis of clinical progression only. Most of these limitations arise because scintigraphy measures the osteoblastic reaction caused by the presence of tumour in the bone rather than the metastatic tumour itself. Similarly, the Response Evaluation Criteria in Solid Tumours are of little use for bone disease because isotope bone scanning is linked to the bone reaction to the tumour rather than tumour bulk. By contrast, PSMA PET and CT and whole-body MRI directly assess the tumour and have higher sensitivity for small metastases than scintigraphy does.

Patients and clinicians are driving the increased use of ad-hoc next-generation imaging. Early detection of disease progression could enable patient access to more therapies, including targeted radiotherapy and PSMA-targeted therapies, and could facilitate enrolment in clinical trials. However, data for drugs such as abiraterone were derived from clinical trials in which men had evident disease as detected by conventional imaging, and trial outcomes were based on treatment continuing to progression as detected by such imaging.^126,128,190,191^ Starting or stopping treatment earlier because of more sensitive imaging might not translate to clinical benefit and could increase costs or decrease efficacy.

The development of robust response criteria based on next-generation imaging could allow early detection of drug efficacy and help to address prostate cancer heterogeneity. Although emerging criteria for acquisition and interpretation of next-generation imaging, such as PSMA PET progression^192^ and MET-RADS-P,^193^ have been suggested, validation is required. There is thus a crucial need to include next-generation imaging in parallel with conventional imaging when undertaking clinical trials.

### Conclusions

In the past 20 years, new technologies have proliferated in prostate cancer care. The role of these technologies is likely to expand, and care needs to be taken to adequately assess effects on patient outcomes and on cost-effectiveness. The ability to cheaply and rapidly sequence DNA and linked developments allowing use of ctDNA are largely restricted to research settings, except in the context of selection of patients who may benefit from PARP inhibitor therapy. However, further therapies requiring molecular selection are highly likely to become available and use of ctDNA to monitor both tumour response and tumour evolution is likely to become part of clinical practice, at least in HICs. Highly accurate imaging technologies like PSMA PET and whole-body MRI again show great promise for improving decision making but have so far largely been assessed solely in terms of accuracy rather than in terms of effects on clinical outcomes. Widespread use of these technologies also makes interpretation of the evidence base developed before their advent more complex. Although the improved accuracy is attractive, trials still need to integrate both conventional and next-generation imaging to bridge the gap between old and new evidence and to enable cross-comparison. Evidence that better imaging improves clinical outcomes remains lacking and producing such evidence needs to be a key focus of future studies. New developments, such as radioligand therapy closely linked with PSMA PET, again point to exciting new therapeutic approaches.

## Part 6: The role of education in modifying prostate cancer outcomes

Raising awareness of prostate cancer plays a central role in effective early detection and treatment. If diagnosis and management of prostate cancer is to improve globally (and especially in LMICs), then education about the disease will be important. A pan-cancer systematic review^76^ that included six prostate cancer-specific studies identified low health literacy as a major barrier to early cancer diagnosis in LMICs, alongside the stigma of a cancer diagnosis and restricted access to primary care, particularly in rural areas. Banerjee and Kaviani^75^ suggest that low public awareness about prostate cancer correlates with increased incidence of advanced disease and mortality in sub-Saharan Africa. Awareness is highly variable within countries, but generally is associated with overall level of education, with only 2·9% of people with no formal education being aware of prostate cancer in one study.^194^

### Information and education as tools of change

Access to reliable, balanced, and up-to-date health information both at the personal and systems levels is key to effecting improvements in care and to support patients and their families in clinical decision making. The rapidly changing landscape of communications technologies and AI presents opportunities for positive change. According to the International Telecommunication Union, in 2015, there were more than 7 billion mobile telephone users globally.^195^ In almost half of the countries with available data for 2018–20, more than 90% of the population owned a mobile phone and in another ten countries the figure was greater than 80%.^196^ In only three countries did less than 50% of the population own a mobile phone (lowest proportion 45%). The global proliferation of mobile devices and growing access to high-speed internet even in remote locations in LMICs is changing the way health information is collected and accessed. Digital solutions delivered through mobile health apps have the potential to transform prostate cancer care by increasing public awareness, providing accurate, comprehensible information to patients, and improving shared decision making.^197^ Public awareness and information will be increasingly important for risk-adapted early detection of prostate cancer, and there is growing interest among health-care providers, cancer charities, research funders, and public health bodies in disseminating effective and culturally sensitive information via apps.^198^ Ensuring the accuracy and comprehensibility of this information is crucial.^198^ Use of mobile phone as a tool for dissemination of educational material about prostate cancer has only been assessed in high-income English-language settings.^198^ Meanwhile, survey results^199^ published in 2023 suggest that eight in ten US Adults access online information about prostate cancer—YouTube and TikTok were frequently consulted sources. Although there is high-quality content about prostate cancer on social networks, there is also a risk of exposure to misinformation. A study^200^ of the top 150 YouTube videos about prostate cancer found that 115 (77%) had some type of biased, commercial, or misinformative content in the video itself or the comments section. For example, a greater proportion of videos described benefits of treatment than the associated harms. Many videos also contained outdated information. There are also concerns about the use of unregulated health apps and international agreement on minimum standards of quality assurance are needed,^201^ including regulations to ensure privacy, security, interoperability, clinical safety, accessibility, and inclusion.^202,203^

An emerging use for mobile phones is the measurement of PSA. Barbosa and colleagues^204^ described portable smartphone camera-based quantification with a fluoropolymer-based microfluidic device that can provide reliable PSA measurements using a device that analyses a pin prick of blood. A smart phone camera can then be used to generate a reading. Near-patient point-of-care technologies are likely to become much more prevalent in all health settings in the future.

### Patient-centred approaches

WHO has formulated a framework for improving cancer care in LMICs.^205^ A key component of the framework is continuity of care. For chronic diseases like cancer, continuity of care is complex because it involves a range of health-care professionals in patient care. In HICs, there is strong evidence for the effectiveness of patient-held records, mostly related to older paper-based record systems.^206^ Data are scarce in LMICs, but a 2021 review^206^ suggested that health-care providers in LMICs perceived that use of patient-held records improved the availability of medical information from other providers. This review suggested that, for all patients, patient-held records provided reliable information about their health condition. Rapid, full access to personal electronic health records is key to empowering patients to manage their health and collaborate with health-care professionals.^207^ The COVID-19 pandemic hugely increased patient and clinician access to electronic records and to apps (eg, the NHS app, which gives all UK citizens instant access to many aspects of their health records such as their medication and vaccination records and outpatient appointments). The UK NHS Patient Portal also gives access to health records and allows patients to request medicines and consultations.

Medical records that interface with guidelines by offering context-sensitive advice (eg, software that, rather than linking to a generic set of guidelines, identifies the correct part of the guideline for an individual and links directly to that) could be extended to patient-held records. More extensive access to electronic patient records is generally being widely implemented in HICs. Apps such as the Zoe COVID app show how public health information can be collected by non-governmental organisations directly from patients. Websites such as the NHS’s Predict Prostate allow users to enter their details and disease parameters to get personalised data about likely outcomes, including cancer control, competing risks of death, and probable toxic effects of treatment based on matching to similar patients included in large randomised trials like ProtecT.^6^ Giving patients access to their medical records and to AI-based support tools puts them in a proactive position with respect to making evidence-based decisions about treatment. These support tools can be tailored to reflect the resource setting in which the patient is being managed.

Challenges to progress include the development and sharing of the requisite software systems, issues around information governance, data protection, and data sharing with unauthorised third parties, and assurance of data security. Neither governments nor large media and technology companies are widely trusted by the general public, often for well founded reasons, as evidenced by controversies around election interference, government 5G contracts, and claims of so-called fake news, etc.

### Education of medical personnel

Education of medical professionals is required at several levels. Basic properties of cancer (in general) and prostate cancer (in particular) should be covered in more detail in medical, nursing, pharmacy, and other health profession curriculums. The curriculum should include recognition of signs and symptoms, diagnostic workup, the principles of surgical treatment, radiotherapy, and systemic therapy, and methods for controlling symptoms, including palliative care. This education needs to be coupled with training addressing stigma surrounding intimate examinations, which can be a barrier to clinicians implementing their learning in practice.^208^

Education of primary care physicians, public health nurses, and other front-line health professionals (including pharmacists in some LMICs) about prostate cancer is essential, so that they can in turn educate patients to be aware of signs and symptoms of the disease (including advance disease) and to seek medical attention.^209^ Furthermore, these primary medical contacts should specifically aim to provide education to men at high risk, such as those with a strong family history, about the importance of early detection of prostate cancer. Doctors of all disciplines who treat the disease should be educated about optimal management and adaptation of management in resource-strained environments. Education should also emphasise how to modify treatment for elderly populations, in view of the demographics of prostate cancer.^210^ Oncologists should be trained to critically review evidence from clinical trials. Development of clinical trials expertise is particularly needed in LMICs, so that trials relevant to specific regions can be designed and implemented.^211^

General medical education in LMICs is often of high quality but can be more variable than that in HICs—for example, UK hospitals maintain a list of medical schools globally from which degrees are not recognised for practice in the UK.^212^ There are training courses sponsored by American Society of Clinical Oncology, the European Society of Medical Oncology, and other organisations to improve the education of oncologists in LMICs in evidence-based medicine and in understanding and participating in clinical trials. The American Society of Clinical Oncology’s International Education Study Group runs several programmes. There are courses that aim to increase general cancer knowledge among primary care physicians, multidisciplinary cancer management courses that focus on management of particular types of cancers, and courses focused on palliative care and supportive management. There are also international clinical research courses focused on teaching about evidence-based cancer management and clinical trials. Organisations in LMICs can apply to host 2–3-day workshops, with the American Society of Clinical Oncology providing organisational support and funding around three international experts to join local faculty in teaching. These courses have taken place in many countries, including Argentina, Brazil, Chile, China, Colombia, Greece, India, Morocco, Romania, Russia, South Africa, Türkiye, and Uruguay. Practitioners and trainees from LMICs that participate in these educational events are knowledgeable and provide evidence-based care, but there is substantial self-selection. Meaningful collaborations between HICs and centres in LMICs are to be strongly encouraged to facilitate back and forth transfer of ideas as well as providing training.

Some courses are moving to a virtual learning format, which might allow for greater dissemination of information. The American Society of Clinical Oncology also has a virtual mentoring programme that connects mentees in various countries with an appropriate mentor. It also collaborates with Health Volunteers Overseas to provide visiting oncologists to teach in LMICs. Other organisations have similar programmes, and many large cancer centres in high-income countries have established partnerships with institutions in LMICs to facilitate exchanges of staff and trainees and to provide education.

Additionally, regional research collaborations, such as the African Organisation for Research and Training in Cancer, are platforms for providing training and advocacy, and act as a forum for research focused on local patient needs. These organisations can also drive international collaborations, such as the Men of African Descent and Carcinoma of the Prostate Consortium in collaboration with the American Society for Preventative Oncology,^213^ and the Prostate Cancer Transatlantic Consortium, which links the Mayo clinic with 20 sites in Nigeria.

New models of care that distribute tasks to suitably trained non-medical staff are more likely to be implemented and succeed than solutions that depend on doctors—particularly in LMICs, but also in HICs, where training of staff such as physician assistants should become more widespread. For such programmes to be robust and safe, tasks need to be broken down and rebundled according to complexity, allowing fully trained doctors and nurses to work at the top end of their skill sets. Prostate cancer diagnosis is amenable to such task shifting. The basic initial diagnostic requirements are for PSA testing and biopsies. The PSA test can be provided by mobile point-of-care machines no larger than a desktop printer. The technologies are like the lateral flow antigen tests widely deployed for COVID-19 testing, but with a quantitative machine readout. Readout can be either via a dedicated machine, or can utilise external calibration, such as a mobile phone.^204^ Choice of device will depend on a range of factors, including the need for portability, cost, and convenience. Such devices require minimal set-up and maintenance, no laboratory infrastructure, and minimal training to operate (and thus do not require extensive medical training), and can be fitted in mobile clinics. Transperineal biopsy can also be provided in similar settings with transrectal ultrasonographic guidance, and can be safely nurse-led rather than performed by physicians.^214^ Key skill sets include obtaining the biopsy sample (and preventing complications from doing so) and immediate tissue processing to prevent tissue degradation: biopsies need to be fixed, cut, and stained, for which suitably trained and equipped staff are required. AI-based pathology reading systems are developing fast and could help to complete the diagnosis.^73,74^

If this task-shifting is implemented, diagnosis could be largely nurse-delivered or physician assistant-delivered with key elements (such as pathology sample processing) provided by technicians. The main role of doctors would be in supervision and quality assurance (eg, for pathology) or in providing key aspects of treatment, such as surgery. Numerous commercial cloud-based patient record systems already exist. Many include inbuilt analytical tools and AI-linked speech-recognition systems. Systems that use AI to generate discharge letters performed as well as junior doctors at this task in one study.^215^ It is easy to envision that decision making could be heavily supported by AI-based systems working on the cloud-based systems, which can link with AI-based information systems that can health professionals with appropriate advice and information. Commercial systems with this functionality already exist and are likely to become increasingly widely available.

### Education of the general public

It is important for the general public to recognise key features of advanced prostate cancer: the fact that the disease is common in older men, its major symptoms (often bone pain due to metastatic spread), and that treatments are available (including inexpensive and simple ones such as hormonal therapy) that can prolong survival and decrease suffering. There is no known way of preventing prostate cancer so the best way to mitigate harm is through early diagnosis. Multiple channels of communication are needed, including social media, television, radio, and newspapers, and celebrities trusted by the public and community hubs must be engaged to ensure maximum reach for such education.^216,217^ Evidence suggests that celebrities addressing their health concerns^216,218^ and information provision in male community hubs such as barbershops^217^ can help to effectively spread awareness. Most educational studies focus on short-term knowledge gains as the primary outcome, but there is little to no assessment of whether such knowledge gains affect clinical outcomes. Thus, there is a need to develop studies that assess the effect of education on clinical outcomes.

Public awareness of prostate cancer varies between LMICs, ranging from 97% in a Brazilian cohort^219^ to less than 50% in many African nations.^220–225^ Various studies have assessed knowledge of, attitudes towards, and perceptions of prostate cancer between countries, but because different methods were used comparison is difficult.^220–225^ Studies of public awareness of prostate cancer^78,219^ in a range of countries in Sub Saharan Africa suggest widespread misconceptions about prostate cancer symptoms and treatment, coupled with reliance on traditional healers and taboos around discussing diagnoses. However, these issues are not confined to LMICs—false or inaccurate information also circulates in HICs, including on YouTube.^200^ Hence there is a great need to increase prostate cancer awareness among people with little formal education and low literacy. Approaches could include the use of visual scoring systems to explain symptoms and assess patients. For example, patients with low educational level can reproducibly complete the Visual Prostate Symptom Score for assessing lower urinary tract symptoms without assistance.^222^ Other examples include the *Cancer & You* booklet produced by Global Oncology, which is available in several African languages with visual aids for patients with low literacy levels.^223^ Experience in South Africa also highlights the challenges patients face in navigating complex health-care systems and understanding the implications of treatments.^221^ The American Cancer Society has worked with local organisations to develop culturally appropriate educational materials for east African audiences (in Ethiopia, Kenya, and Uganda) about cancer, and has received a grant from the Merck Foundation to create a comprehensive patient navigation programme with a development and implementation guide that will be piloted in health institutions in Asia and Latin America.^226^

Community outreach efforts, such as those of the Ugandan Cancer Institute, should also be used to educate the general public in LMICs about prostate cancer. The Ugandan Cancer Institute used an asset-based community-development approach that was sustainable and gained community buy-in, and that was combined with clinician-led educational events and a television and radio campaign. This programme has led to increased uptake of breast and prostate cancer detection in rural communities.^224^

Use of patient navigators can be coupled with education efforts. Introduced in Harlem (New York, NY, USA) in the 1990s to reduce barriers to care for breast cancer, patient navigators are community support workers who help patients to find their way through complex health systems involving multiple specialists and provide general awareness and education. Patient navigators can address many issues faced by patients in attempting to understanding their treatment. Evidence shows the effectiveness of patient navigators in reducing time to care (a surrogate for improved outcomes) for patients with prostate cancer in the USA,^225^ but no prostate cancer-specific studies have been done in LMICs (although use of patient navigators in LMICs can improve compliance with screening programmes and improve outcomes in other cancers^227^). Non-governmental organisations, such as Project PINK BLUE in Nigeria, are piloting such initiatives (panel 6). Patient navigation studies have not been done in the poorest populations^229^ with the greatest educational need, and patient navigators are only effective if patients can afford treatment. Bukowski and colleagues^230^ reviewed the practicalities of implementing patient navigator initiatives in LMICs, and identified data for the effectiveness of the approach but also barriers to implementation. They recommended a three-fold strategy of targeting gaps in infrastructure, using a customisable protocol and training for navigators, and engagement with policymakers. Jatho and colleagues^231^ showed that increased primary care capacity can be successfully combined with educational initiatives (ie, help with identifying problems and accessing care) about prostate cancer in Uganda to improve early detection.

### Social media

Social networks have created new channels for global dissemination and knowledge exchange about prostate cancer.^232^ The use of social media is increasing globally, with approximately 3·6 billion users in 2020.^233^ Social media could be a source of reliable education and support for patients with prostate cancer and their families. For example, there are private groups on Facebook for prostate cancer survivors who share common features, such as a similar stage of disease or treatment selection. There are also large online health communities where patients and their families interact with each other. These groups can be used by patients to share their experiences or their perceptions of health-care providers.^234^ Research has suggested that participation in an online network could influence prostate cancer treatment decisions.^235^

Most major medical conferences encourage participants to share highlights on social media through the use of dedicated hashtags.^236^ Other social media discussions provide opportunities for patients and their families to engage with health-care professionals and scientists specialising in prostate cancer. For example, there is a social media-based prostate cancer journal club (indexed as #prostatejc via X), which is held monthly to discuss important new research articles.^237^ Historically, journal clubs were limited to members of one department and were not open to the public. Social networks are breaking down barriers to information and facilitating global interdisciplinary sharing of information.

### Education of government public health departments

Government public health departments need to be made aware that the incidence of prostate cancer will increase with rising life expectancy, and that funding of measures to increase awareness of the disease and associated treatment is important. Such education requires both reliable registry data to define the scale of the problem, and robust evidence about which educational and management interventions are most cost-effective.

Governments public health departments might be aware of the cancer scale and profile in their countries through the Global Cancer Observatory and related papers,^238^ as well as other published summary statistics (eg, the Global Burden of Disease reports).^5,75^ Data are needed to clarify the most effective and cost-effective educational interventions, which governments then need to prioritise and couple with other initiatives (such as training) that widen access to diagnosis and treatment of prostate cancer.

### Conclusions

The rapidly changing landscape of modern communications technology offers huge opportunities to connect to people and to educate about prostate cancer. Even people living in remote places often still have access to mobile telecommunications. Social media channels like TikTok, Instagram, and X have huge reach. Popular influencers could be a novel way to disseminate health information to people. Health-care providers should exploit these channels to link people to more conventional information sources and active case-finding programmes. Health checks linked to diagnostic programmes could also be promoted. Furthermore, smartphones have huge interactive capabilities that could be exploited. Mobile patient-held smartphone records are a key new tool with huge potential to drive change. Although these channels are also open to misuse and misinformation, if appropriately managed, there are huge opportunities to effect positive change.

## Part 7: How to bring about the required changes to prostate cancer care

### Survivorship

Most men treated for non-metastatic prostate cancer in HICs do not die from prostate cancer. For example, in the UK, around 50 000 men are diagnosed with prostate cancer annually and around 12 000 men die from the disease (around 7000 of whom were diagnosed with metastatic disease).^26^ Overall, 78% of UK patients survive 10 years or more.^26^ The data for other HICs are similar. Therefore, in HICs, men often live for many years—even decades—from diagnosis, with the consequences of treatments such as surgery or radiotherapy, however.

Prostate cancer follow-up is mainly based around monitoring of PSA concentrations in both HICs and LMICs. Regular hospital attendance is thus unnecessary: PSA checks can be provided effectively via primary care and can be tracked by the patients themselves if suitably informed and motivated. Disease symptoms and treatment side-effects can be managed by nurses or physician assistants.

Many men will also have long survival with advanced disease: with modern treatment in HICs, median overall survival in patients with metastatic prostate cancer (factoring in stage migration from increased use of PSMA PET to detect metastases) is 5–7 years and rising.^120–128^ Most of these men will be living with the side-effects of long-term androgen deprivation. Some will be of working age, and should be encouraged to stay economically active, which benefits both them and society by improving family finances, often in settings where men are the main earners.

In LMICs, where prostate cancer is more likely to be metastatic, men are likely to be on hormone therapies long term. As their cancers worsen, they will need additional therapies, including other drugs, and where available, radiotherapy and surgery for complications like bladder outflow obstruction. Navigation of the complexity of relapsed prostate cancer care is a situation in which smartphone-based assistance could supplement care provided by local health-care professionals: patients could get help with navigating care and managing side-effects and health-care professionals could be supported in their delivery of appropriate treatment. For both patients and professionals, accessible electronic records could be transformational and are a key health-care priority. Improved care can have large economic benefits both for the patient (by keeping them economically active and independent) and for society (better care is likely to lead to better outcomes and fewer catastrophic events with attendant costs). For example, trials of intensified androgen deprivation therapy for metastatic disease show long-term reductions in serious complications.^120–128^ Studies of both abiraterone and docetaxel show that such reductions are cost-effective at a societal level.^239–241^

Frequently in LMICs (and some HICs—notably, the USA), patients have to co-pay (or indeed pay in full) for their care. The proportion of people with cancer in the USA who are not fully insured varies with factors such as age, income, and location. According to the American Cancer Society, in 2021, roughly 11% of adults aged 18–64 with cancer were uninsured and another 11% were underinsured.^242^ Globally, more equitable health care systems have been estimated to potentially save 8 million lives annually, with large societal and economic benefits.^241^ Systems that guide patients to optimal care and support them through treatment are likely to be money saving and could lead to improved outcomes and better control of side-effects. An illustrative example is guiding patients to undergo orchiectomy in place of GnRH analogue injections for long-term androgen deprivation in instances where patients are paying out of pocket for their care (which would save them large amounts of money).

### How to ensure that guidelines are followed

Many treatment guidelines are available for prostate cancer, and are usually published as standalone documents. A major problem is the time needed to access and identify which parts of a guideline are relevant to a given patient. There is a need to integrate patient records with guidelines, thereby allowing for individualised recommendations. Computer-interpretable guidelines that interface with electronic health records are therefore likely to be a growth area. Companies are developing such links to offer so-called patient-like-me data for improved individualisation (eg, Predict Prostate).

AI-based interfaces have the potential to enable guidelines to become dynamic and tailored to individuals rather than static, difficult-to-access, and often-incomprehensible reference documents. A growing volume of data suggests that AI-based pathology and radiology systems perform well on routine tasks. Such systems could help to democratise health care, empower patients, and improve the care given by health professionals. A growing debate in AI research is whether work should focus on using AI for medical tasks, such as interpreting pathological specimens, or used to find hidden features not apparent to the human eye, although it is likely that these two approaches are complementary. They could provide routes for direct patient input into care delivery and offer new ways to improve patient and public involvement. Supporting patient and public involvement in this way should become a high priority for funders of care because it provides a route to improved services that are not dependent on training (scarce) additional medical or related staff. Input will be needed to ensure that AI-based systems can perform reliably when care decisions are dependent upon them. Also, when AI is used to replace a human—for example in the generation of medical letters^215^—quality and accuracy checks will be essential. Buy-in from health professionals will be key to the success of these changes and targeted education will be required. However, in a world of growing unmet health-care needs, rapid solutions to allow trained staff to focus on the key parts of their jobs and not have their time monopolised by more mundane tasks are highly attractive.

Large improvements in prostate cancer outcomes would probably result if health professionals followed high-quality guidelines (such as those produced by the American Society of Clinical Oncology, European Association of Urology, the American Urological Association, and the European Society for Medical Oncology) and ensured that patients receive the right treatments at the right time. Research into implementation science deserves more attention and funding. Simple interventions to encourage knowledge transfer, such as audit and feedback, have been effective in encouraging change in physicians’ behaviour.^243^ There continues to be fixation on new research, which can lead to overlooking substantial improvements that could be made in terms of patient outcomes if existing knowledge were well implemented.

## Conclusion

The recommendations that we make in this Commission could help to improve the diagnosis and management of men with prostate cancer worldwide. Governments and health-care funders should invest resources into immediate implementation of evidence-based diagnostic tools and treatments within their sphere of operations to address adverse outcomes for men with prostate cancer. Furthermore, in view of the large projected rise in cases, long-term changes need to be rolled out from now if large increases in prostate cancer deaths are to be prevented. Here and in panel 7, we detail four key actions that need to be taken, with tailored recommendations for HICs and LMICs.

First, diagnostic pathways in all health-care settings need to be improved to facilitate early detection of clinically significant prostate cancer. These pathways need to be integrated with diagnostic approaches for other common men’s health issues. Avoiding overdiagnosis and overtreatment while still reducing the frequency of late-stage diagnoses is essential in HICs. In LMICs, meanwhile, the overwhelming issue is late diagnosis, and different solutions are proposed (as stressed earlier, there are no public health measures that are known to prevent prostate cancer and harms can be mitigated only by earlier diagnosis). Second, accessible electronic medical record systems should be developed to improve access to health information for patients and to facilitate use of AI to allow better interpretation of available data, which could complement or supplement deficits in health profession numbers and skills. More than half the population of Africa has no access to cancer care.^7–9^ By contrast, at least 70% of the adult population has access to a smartphone (and this number is rapidly rising).^196^ Smartphone-based medical apps and medical records systems are rapidly becoming widely available (eg, the NHS app), and this approach needs to be urgently extended to LMICs to empower patients. Establishment of smartphone-based health services in LMICs has huge potential to broaden access, improve education, and gather public health data on disease patterns. Such systems are already in widespread use in HICs, and the challenge in LMICs is to roll them out in affordable ways.

Third, pragmatic recommendations for diagnosis and treatment, tailored to national resource levels and patterns of disease, should be implemented. The amount of effective care that is affordable will vary by country and region according to disease patterns and income. Countries should seek to establish either national or regional guidelines for minimal care based on international guidelines plus other resources such as the WHO list of recommended medicines, which already includes drugs such as abiraterone and docetaxel. A template for care for metastatic disease is included in the table.

Finally, research into risk-stratified regulatory models, in which the level of regulatory burden is related to the risk of the change under assessment, should be supported by governments. At present in HICs, regulations for trialling existing drugs in novel settings attract similar levels of monitoring and scrutiny to licensing trials of new drugs. Research to assess a new indication for an established drug with a known safety profile, for example, should not require the same level of documentary and trial support as first registration of a novel chemical entity. Other examples of research that would benefit from a risk-stratified model include cost-effective diagnostic methods, repurposing or dose de-escalation of drugs, and better understanding of the effect of ethnic differences on outcomes. Current regulations inhibit trials of older, off-patent drugs that could potentially have huge value in all disease settings, not just prostate cancer. Clinical research is dominated by pharmaceutical company-sponsored trials, with the aim of developing and marketing new drugs in HICs. Such trials often bring only marginal gains at very high cost. Key areas of research for transnational institutions such as WHO should include attempting to clarify the role of existing, off-patent drugs and repurposing drugs (eg, abiraterone) for use in prostate cancer. Potentially easy wins are available from dose de-escalation trials, where either the dose is reduced or frequency of administration is decreased. If no reductions in efficacy occur, then the costs associated with treatment drop and side-effects potentially decrease. Low-dose abiraterone is a good example of dose de-escalation in prostate cancer.^144^ The biggest potential gains for both patients and governments are related to achieving early diagnosis—an area of little pharmaceutical interest. Integration of trials with technologies such as smartphones offers opportunities for increasing the number of patients diagnosed early. Research into, and knowledge about, the biology of prostate cancer is heavily focused on White men. Less is known about the biology of prostate cancer in non-White populations, despite ethnic differences in incidence and outcomes both internationally and within immigrant populations in HIC. Interventions like screening and active surveillance have not been assessed in non-white populations and the risk–benefit ratios could differ substantially in these populations.

Prostate cancer is not preventable. The only effective way to mitigate harm is to implement strategies for early diagnosis and effective treatment. Implementation of our recommendations (panel 7) is urgently needed and will substantially counteract the coming increases in prostate cancer.

## Figures and Tables

**Table:** Options for treating metastatic prostate cancer in low-income and middle-income countries and in high-income countries

	First-line therapy for hormone-sensitive disease	Possible additional first-line therapies	First-line treatment of relapsed disease	Second or subsequent relapse	All relapse settings
Low-income and middle-income countries	Androgen deprivation therapy with gonadotropin-releasing hormone analogues with offer of orchiectomy either at initiation or once established on treatment. All patients should be given bone-protecting agents (eg, bisphosphonates, calcium, vitamin D) to prevent bone loss.	Abiraterone commenced at diagnosis (savings from orchiectomy can be used to offset costs or generic abiraterone) or alternatively six cycles of docetaxel. As generic drugs become available, other new-generation anti-androgens, such as enzalutamide, could be used. Radiotherapy to the prostate (only if disease burden is low).	Up to ten cycles of docetaxel (or abiraterone or other new-generation anti-androgens).	As per first-line treatment of relapsed disease	Good-quality surgical and radiotherapeutic palliation, opioid analgesics. Bone-protecting agents increasingly important as survival times rise.
High-income countries	Androgen deprivation therapy with offer of orchiectomy either at initiation or once established on treatment. All patients should be given bone-protecting agents (eg, bisphosphonates, calcium, vitamin D) to prevent bone loss.	New-generation anti-androgen therapy (eg, abiraterone, enzalutamide, apalutamide and darolutamide); choice of agents depending on local funding arrangements. For younger, fit patients with high-burden disease, both docetaxel and a new-generation anti-androgen might be indicated. Radiotherapy to the prostate (only if disease burden is low).	Up to ten cycles of docetaxel (or up to ten cycles of cabazitaxel if patient has already received docetaxel). Radium-223 (for people unable to undergo chemotherapy unfit and people with bone-predominant disease) PARP inhibitors (dependent on DNA damage repair mutation status).	Prostate-specific membrane antigen-targeted lutetium, radium-223, PARP inhibitors, cabazitaxel. Pembrolizumab in patients with high microsatellite instability (about 3% of patients) in the USA and some European countries.	Good-quality surgical and radiotherapeutic palliation, opioid analgesics. Bone-protecting agents increasingly important as survival times rise. High-dose metastasis-directed radiotherapy likely to become increasingly important

## Key messages

- We project that the number of new cases of prostate cancer annually will rise from 1·4 million in 2020 to 2·9 million by 2040. Changing age structures and improving life expectancy are predicted to drive big increases in the disease.
- The projected rise in prostate cancer cases cannot be prevented by lifestyle changes or public health interventions.
- Late diagnosis of prostate cancer is widespread worldwide but especially in LMICs, where late diagnosis is the norm. The only way to mitigate the harm caused by rising case numbers is to urgently set up systems for earlier diagnosis in LMICs. Trials of screening are urgently needed in LMICs to better inform ways to improve early diagnosis.
- Early diagnosis systems will need to incorporate novel mixes of personnel and integrate the growing power of artificial intelligence to aid interpretation of scans and biopsy samples.
- As the rise in prostate cancer is likely to be mirrored by rises in other conditions such as diabetes and heart disease, early diagnosis programmes should focus not just on prostate cancer but on men’s health more broadly.
- Outreach programmes are needed that harness the broad global availability of smartphones as tools for education about prostate cancer (using both social media and traditional media), as are programmes that assist people with navigation of health-care systems.
- Most prostate cancer research has disproportionally focused on men of European origin, despite rates of prostate cancer being twice as high in men of African heritage. Better understanding of drivers of ethnic differences in prevalence of the disease is a key research priority.
- Treatment of advanced prostate cancer remains a problem, and affordable therapies are available but are unevenly distributed. Consistent use of these therapies is a cost-effective way to reduce harm from prostate cancer.
- There remains a shortage of specialist surgeons and radiotherapy equipment in LMICs, and addressing this shortage is key to improving prostate cancer care globally.

LMICs=low-income and middle-income countries.

## Panel 1 Focus on men of African descent

Data are insufficient to identify the driving factors of the disparately high incidence of prostate cancer in men of west African descent. Comparing incidence and mortality patterns among such men with those among men of other races and ethnicities could help to better define the relative contributions of social and environmental factors, race, ethnicity, ancestry, genetics, epigenetics, and access to care to these disparities. In south Florida, Hispanic men have a nearly 100% increased lifetime risk of developing prostate cancer and are twice as likely to die from the disease than non-Hispanic white men—a disparity that is not apparent nationally.^30^ US census data show that Hispanic people in south Florida are more frequently of Caribbean descent and are more likely to have west African ancestry and identify as Afro-Latino than the Hispanic population in the rest of the USA.^31^ Furthermore, prostate cancer mortality is similar in Caribbean and Hispanic populations in south Florida,^30^ suggesting a common cultural, environmental, or ancestral link. More comprehensive global registry data, particularly from Africa, could shed light onto the potential causes of global disparities and heterogeneity in prostate cancer risk across populations.^5,16^

## Panel 2 Focus on India

Prostate cancer accounts for 3% of all cancers in India, with an estimated 33 000–42 000 new cases diagnosed annually.^32,33^ The age-standardised incidence is 4·8 cases per 100 000 population per year; incidence has increased by about 30% nationally and by 75–80% in urban populations in the last 25 years.^32,33^ As a result of low awareness of prostate cancer and little systematic testing of PSA, most men present with metastatic disease.^32,33^ Although estimation of mortality has been challenging because of under-reporting of cancer-related deaths and the omission of cancer site in death certificates, about two-thirds of patients with incident disease die. A unified national cancer patient database collating data from multiple registries, stored in a public electronic repository, would build the foundation for understanding the burden of prostate cancer in India and would be useful for estimation of trends, identification of missing data, and informing policy decisions.

## Panel 3 Recommendations to improve diagnosis of prostate cancer

**High-income countries**

- PSA-based early detection programmes linked with MRI should be implemented for men at high risk of death from prostate cancer:
  - aged 50–69 years with a least one other risk factor based on an individualised risk-adapted early detection strategy
  - aged 45 years or older with a family history of prostate cancer
  - aged 45 years or older and of African heritage
  - aged 40 years or older with *BRCA2* mutation
- Studies incorporating upfront MRI or calculation of polygenic risk scores, or both, alongside measurement of PSA concentrations are needed to further improve detection while mitigating the risk of overdiagnosis
- PSA detection-based programmes should be explicitly linked to increased utilisation of active surveillance to mitigate the risk of overtreatment of indolent disease
- Sociodemographically targeted education and early diagnosis programmes in high-risk populations could help to reduce the proportion of people who present with advanced disease

**Low-income and middle-income countries**

- Diagnostic capabilities and education should be expanded to raise awareness and increase early diagnosis
- Education, screening, and detection programmes for multiple diseases (eg, hypertension, diabetes) should be combined as an intervention package; education should be targeted at high-risk groups
- Targeted case-finding in high-risk groups are required
- Novel strategies to empower patients, such as cloud-based patient-held records and mobile testing with a broad focus on men’s health, should be urgently explored

PSA=prostate-specific antigen.

## Panel 4 Radiotherapy access in Brazil, India, and Africa

**Brazil**

Brazil has high income disparity but also has a comprehensive publicly funded health-care system: the Sistema Único de Saúde. A third of the population has health insurance and thus receive private care roughly equivalent to that in high-income countries. Care for those relying on the Sistema Único de Saúde is patchier: although ostensibly comprehensive in its coverage, there are substantial geographical variations in, for example, access to radiotherapy equipment. Of the 439 health regions in the country, 329 (75%) do not have radiotherapy machines, and 307 (70%) do not have radiation oncologists.^109^ The Brazilian Society of Radiotherapy estimates that around 100 000 patients annually who might benefit from radiotherapy did not receive it, and Mendez and colleagues^109^ estimated that this lack of access results in around 900 avoidable deaths each year from prostate cancer alone.

Under the 2012 Sistema Único de Saúde Radiotherapy Expansion Plan, the Brazilian Ministry of Health contracted with a private company to purchase and supply 80 linear accelerators for the public system, and to establish a training and capacity-building centre. Importation of high-tech equipment, parts, and software is associated with substantial difficulties, however, which are exacerbated by inflation and currency depreciation. By 2018, only 13 new linear accelerators had been installed, but the programme has been more successful in the education of medical physicists and radiotherapists: about 160 radiotherapy technicians and 11 medical physicists are now expected to graduate annually.^110^ Schemes like this one, although only partly successful, point towards how capacity in radiotherapy can be achieved when there is political will.

**India**

Historically, cobalt-60-based machines have been used to provide radiotherapy in India. Switching to linear accelerators is associated with issues (as per in Brazil) but improves quality and throughput and reduces the risk of accidents that could result in undesired radiation emissions (because the active radioactive source is replaced with an electrical device that produces no intrinsic radioactivity). However, access to radiotherapy has grown substantially, from 35 linear accelerators and 290 cobalt-60 units in 2001, to 656 megavoltage units in 2019,^111^ of which around 95% can deliver intensity-modulated radiotherapy. In a survey^111^ of 88 radiation oncologists, who were selected to represent centres from across all areas of India, 82 (93%) report using intensity-modulated prostate radiotherapy. They also report delivering prostate radiotherapy at one proton therapy unit. WHO recommends one megavoltage radiotherapy unit per million people. To meet this recommendation, India would need an additional 600 or so units to ensure that the 800 000 people with cancer who require radiotherapy each year^112^ can be adequately treated.

The growth in radiotherapy access that has happened so far has been concentrated in urban areas, and mostly serves wealthier people. Although coverage of modern radiotherapy treatments within governmental health schemes has improved access overall, the poorest sections of the population have little access even to palliative radiotherapy.^111,112^

**Africa**

29 African countries, with a combined population of around 316 million people (ie, 26% of the continent’s total population), have no access to radiotherapy.^7,8^ In most other African countries, radiotherapy availability is scarce and often unaffordable.^7,8^ Until recently, Nigeria, for example, had only eight government-funded radiotherapy machines for more than 200 million people, although plans for expansion are in place.^113^ The scarcity of radiotherapy equipment is most prominent in low-income countries (ie, those with gross domestic products of US$285–1000 per person).^114^ Patients need to travel long distances, potentially involving days of travel, to access the little radiotherapy capacity available in most African countries, which increases cancer mortality. Problems with increasing access to radiotherapy include not only the high cost of implementation, but also maintenance of radiotherapy services and inconsistent power supplies.^1^ Most patients pay out of pocket to access treatment, with little or no health insurance coverage, which further restricts access.^1^ Brazil provides a potential delivery model that could be aspired to. Additionally, regional radiotherapy hubs could provide a bridge to delivery that allows the build-up of expertise.

## Panel 5 Availability of prostate cancer drugs in Brazil, India, and Africa

**Brazil**

In Brazil, the publicly funded Sistema Único de Saúde funds long-established chemotherapies (eg, docetaxel), but newer hormonal agents and newer chemotherapy drugs such as cabazitaxel are not available outside clinical trials. There is a shortage of oncologists in almost half of Brazilian health regions, and almost two-thirds of the health regions do not have rooms for chemotherapy.^129^ The shortage of oncologists and facilities results in patients having to travel, sometimes long distances, so that they can receive appropriate treatment and follow-up. In the private system, patients can be treated according to the US National Comprehensive Cancer Network guidelines (new hormonal agents, cabazitaxel, and radium-223 are all available).^130^ Analysis by this Commission in collaboration with Brazilian oncologists showed that cancer care could be improved by using cost-saving approaches. Orchiectomy costs $85 as a one-off treatment; by contrast, injectable analogues, typically given for a median of 28 months, cost $59 per month when treated via the Sistema Único de Saúde, offering large cost savings with no negative effects on outcomes. The Brazilian Government is examining these approaches.

**India**

In India, allocation of public funding for health care is low (equivalent to roughly US$7 per person^131^) and insurance coverage is patchy, leading to heavy reliance on out-of-pocket spending. The low ratio of oncologists to people with cancer (1:2000) often leads to delivery of systemic therapy by health-care professionals who do not have the requisite specialist training.^132^ The four-tiered system of increasing sophistication for delivery of systemic therapies using the existing health-care infrastructure in India could serve as a template for wider adoption, but whether the necessary political and organisational changes could be implemented remains unclear.^132^ The widespread availability of generics has increased access to life-prolonging agents for advanced prostate cancer, and use of older antiandrogens, oestrogens, and low-dose corticosteroids has decreased.^133^ For patients with metastatic prostate cancer, orchiectomy is offered routinely, which leads to substantial cost-saving.

**Africa**

In most African countries, doctors with specialist training in oncology are rare. People generally pay out of pocket for their cancer treatment, but a very small proportion of people have partial national health insurance coverage.^134^ Wealthy people can often get treatment in private facilities either by paying out of pocket or via private insurance coverage. Failure to complete treatment for financial reasons is common.^134^ A joint initiative by the American Cancer Society and Clinton Health Access Initiative, which started in 2015, has established collaborations with pharmaceutical companies to improve access to cancer care by supplying 20 medications (including docetaxel, bicalutamide, and leuprolide) at affordable prices—about 60% reductions in the standard price. The programme will include Ethiopia, Kenya, Nigeria, Rwanda, Tanzania, and Uganda, which together account for about 44% of cancer cases in sub-Saharan Africa.^135^ The programme also includes a free smartphone app to facilitate patient-led access to affordable cancer care.

## Panel 6 Project PINK BLUE

Project PINK BLUE is a Nigerian non-governmental organisation that aims to challenge public perceptions of cancer and improve cancer care in Africa. With government backing and celebrity support, its campaigns provide an example of how the strategies outlined in Part 6 of this Commission can be combined in low-income and middle-income countries to effect real change. Specifically, to address the shortfall in public in Nigeria of prostate cancer in Nigeria, the organisation launched the programme MEN ON BLUE in 2018, with funding from the Aspire Coronation Trust Foundation. The aim is to promote prostate cancer awareness and screening in urban and rural communities, and to harness the power of social media to increase public awareness. The pilot project focused on three Nigerian cities—Enugu, Abuja, and Lagos—with plans to rolls out the scheme nationwide.

Examples of MEN ON BLUE initiatives include:

- Engagement of community leaders and use of community hubs (eg, exercise centres, places of worship) for screening awareness
- Patient navigation services to connect rural communities to urban hospital centres (based on the US Patient Navigation Research Programme)^228^
- Free telephone advice line
- Free prostate-specific antigen screening (1661 men were screened during the initial pilot project), with results sent via SMS or telephone calls by trained staff
- Psychological support during follow-up after treatment
- Free prostate cancer educational material translated into local languages and disseminated via community medical facilities
- Prostate cancer walks (mass participation sponsored walks aimed at raising awareness and raising funds) coupled to screening recruitment events where men can be offered testing for prostate cancer.

Surveys of MEN ON BLUE participants have identified barriers to continuing engagement with screening and treatment, including financial, educational, and belief-based barriers.^220^ However, overall the project provides a funding and organisational model that could be applied in other low-income or middle-income countries. Many of the issues with prostate cancer awareness are also apparent for other diseases and opportunities to collaborate with other health education and promotion groups are encouraged.

## Panel 7 Key actions and deliverables to improve global prostate cancer care

Action 1: improve diagnostic pathways in all health-care settings to facilitate early detection of clinically significant prostate cancer, and integrate diagnosis into programmes with a focus on broader men’s health issues. Because prostate cancer cannot be prevented, and thus the rise in cases is inevitable, this is the only way to mitigate the rising tide of harm that will result from prostate cancer in the coming years.

- In HICs, pre-biopsy MRI scanning should be used in at least 50% of suspected prostate cancer cases to improve biopsy accuracy and reduce overdiagnosis of low-grade disease within the next 5 years (given that the required technologically is already widely available). Success can be measured via analysis of published public health and insurance data.
- In LMICs, outreach services offering information, prostate-specific antigen testing, and onwards referral for suspected cancer diagnosis and treatment should be established. Success can be measured via analysis of published public health and insurance data (when available).

Action 2: create cloud-based medical record systems to improve access to health information and to facilitate use of artificial intelligence to complement or supplement deficits in health profession numbers and skills. Access to, and control of, medical information allows the addition of tailored advice and assistance with navigation of medical systems. Leveraging the broad availability of smartphones globally and linkage to the extensive online resources (eg, the app produced by IBM as part of a drug-access programme in collaboration with the Clinton Foundation and the American Cancer Society^135^) in a structured fashion has the potential to transform how people in LMICs access health care.

- In LMICs, 25% of men should have access to smartphone-based advice and medical records within 5 years. Suitable software should be made available within the same timeframe by trusted not-for-profit organisations, such as international charities, and should build on existing systems. Data from institutions such as the World Bank and WHO, as well as from relevant software providers, can be used to measure progress.
- In HICs, smartphone health apps are increasingly popular. Their use should be strongly supported: the more control patients have over their health care, the more they can be guided towards better choices.

Action 3: implement pragmatic practice recommendations for maximum benefit, tailored to national resource levels and patterns of disease. Guidelines should be resource-appropriate and should be linked to patient-held record systems so that implementation can be both supported and audited. At present, in HICs with funded health care, evidence shows low levels of compliance with guidelines. Simply consistently delivering what is known to work (and which is funded) is a rapid way to improve outcomes.

- In HICs, 80% of men with metastatic disease should receive intensified androgen deprivation therapy (ie, an anti-androgen in addition to standard hormonal androgen deprivation therapy) within the next 5 years. Progress can be measured via published public health and insurance data
- In LMICs, orchiectomy should be offered instead of gonadotropin-releasing hormone agonists either at diagnosis or once treatment is established in at least 50% of newly diagnosed men within the next 5 years. Men who are already on long-term androgen-deprivation thereapy should be offered the opportunity to undergo orchiectomy and should have the benefits explained. Data from institutions such as the World Bank and WHO, as well as from publicly funded health-care systems (eg, the Brazilian Sistema Único de Saúde) can be used to measure progress.

Action 4: support research on risk-stratified regulatory models, including cost-effective diagnostic methods and repurposing or dose de-escalation of drugs, and on better understanding of the effects of ethnic differences on prostate cancer outcomes.

- Trials should reflect the ethnic mix of the population under study. In international trials, broad geographical spread is a route to better representation of different ethnic. Ethnic and racial representation in trials should be measured by regulatory bodies assessing new applications for marketing authorisations, with a target of 5 years for the introduction of racial targets for trial recruitment by major regulators. In the context of prostate cancer, under-representation of men of African origin, who appear to have distinct disease features, is a particular (but not the only) concern.
- Recording of ethnicity in clinical trials should be mandatory, with inclusion of sufficient non-White participants to allow adequate assessment of both efficacy and toxicity. For ethnicity data, at least 80% of eligible trials should report race or ethnicity data for trial participants within 5 years. Progress can be assessed via public databases such as ClinicalTrials.gov and published trial results.
- In LMICs, at least one trial each of screening, early diagnosis, and treatment should be established within the next 5 years.

HICs=high-income countries. LMICs=low-income and middle-income countries.
